# IdeS, a secreted proteinase of *Streptococcus pyogenes*, is bound to a nuclease at the bacterial surface where it inactivates opsonizing IgG antibodies

**DOI:** 10.1016/j.jbc.2023.105345

**Published:** 2023-10-12

**Authors:** Inga-Maria Frick, Lotta Happonen, Sebastian Wrighton, Pontus Nordenfelt, Lars Björck

**Affiliations:** Division of Infection Medicine, Department of Clinical Science, Lund University, Lund, Sweden

**Keywords:** bacteria, *Streptococcus*, proteolysis, proteinase, IdeS, IgG, DNase, immunity, virulence

## Abstract

The important bacterial pathogen *Streptococcus pyogenes* secretes IdeS (immunoglobulin G-degrading enzyme of *S. pyogenes*), a proteinase that cleaves human immunoglobulin G (IgG) antibodies in the hinge region resulting in Fc (fragment crystallizable) and F(ab')_2_ (fragment antigen-binding) fragments and protects the bacteria against phagocytic killing. Experiments with radiolabeled IdeS and flow cytometry demonstrated that IdeS binds to the surface of *S. pyogenes*, and the interaction was most prominent in conditions resembling those in the pharynx (acidic pH and low salt), the habitat for *S. pyogenes*. SpnA (*S*. *pyogenes* nuclease A) is a cell wall–anchored DNase. A dose-dependent interaction between purified SpnA and IdeS was demonstrated in slot binding and surface plasmon resonance spectroscopy experiments. Gel filtration showed that IdeS forms proteolytically active complexes with SpnA in solution, and super-resolution fluorescence microscopy revealed the presence of SpnA–IdeS complexes at the surface of *S. pyogenes*. Finally, specific IgG antibodies binding to *S. pyogenes* surface antigens were efficiently cleaved by surface-associated IdeS. IdeS is secreted by all *S. pyogenes* isolates and cleaves IgG antibodies with a unique degree of specificity and efficiency. These properties and the finding here that the proteinase is present and fully active at the bacterial surface in complex with SpnA implicate an important role for IdeS in *S. pyogenes* biology and pathogenesis.

Immunoglobulins (Igs) play central roles in the adaptive immune system to mediate elimination of microorganisms that invade the host. The Ig molecule consists of the antigen-binding Fab (fragment antigen-binding) regions that are linked *via* a hinge region to the constant Fc (fragment crystallizable) part. The Fc region initiates phagocytosis by binding to receptors on phagocytic cells, and by binding to C1q, it triggers activation of the classical pathway of complement (1). For a bacterium, it is of crucial importance to modulate host immune defenses to survive. Several bacterial surface proteins showing affinity for Igs have been identified (for a review, see Ref. (2)), and in most cases, these proteins bind to the Fc region of immunoglobulin G (IgG). Two such well-characterized proteins are protein A, expressed by *Staphylococcus aureus*, and protein G of group C and G streptococci (3, 4, 5). The significant human bacterial pathogen *Streptococcus pyogenes* also express IgGFc-binding proteins, and several of these have been characterized (6, 7, 8, 9, 10). Bacterial binding of IgG *via* the Fc region will prevent recognition by the Fc receptor and phagocytic killing. The importance of this Fc binding is emphasized by the fact that genetically and structurally unrelated bacterial IgGFc-binding proteins bind to the very same region in IgGFc (11), a case of convergent evolution suggesting that this binding adds selective advantages in handling host immune responses.

Another bacterial protective mechanism is the expression of proteinases capable of cleaving Igs (for reviews, see Ref. (12)). In the case of *S. pyogenes*, these bacteria secrete IdeS (immunoglobulin G-degrading enzyme of *S. pyogenes*), a cysteine proteinase that is highly specific for IgG (13). *S. pyogenes* causes a variety of infections, including mild throat and skin infections and more severe conditions like necrotizing fasciitis and sepsis (14). Based on variations in a surface molecule, the M protein, *S. pyogenes* isolates are divided into a large number of serotypes (15). IdeS (also known as protein Mac (16)) is produced by all isolates of *S. pyogenes* and cleaves IgG in the lower hinge region of the heavy chain (13). The unique specificity of IdeS, apart from IgG no other substrate is known, is explained by the fact that the proteinase must bind to the Fc region of IgG before cleavage can occur, and the specificity is based on this initial protein–protein interaction (17, 18).

*S. pyogenes* nuclease A (SpnA) is a cell wall–anchored DNase (19). At least eight DNases are expressed by *S. pyogenes* (20, 21, 22), and contrary to SpnA, the other seven are secreted. The *spnA* gene was found in all investigated *S. pyogenes* isolates, suggesting that the DNase is generally present in *S. pyogenes* regardless of M serotype (19). Specific SpnA antibodies were found in higher frequencies in patient serum as compared with serum from healthy donors, indicating that SpnA is expressed during infection (23). It has also been reported that the *spnA* (*spy0747*) transcript is upregulated during growth in human blood (24), and that SpnA promotes bacterial survival in whole blood (23). Furthermore, in blood bactericidal assays and in a murine infection model, a *spnA*-knockout strain was less virulent, suggesting that SpnA contributes to *S. pyogenes* virulence (19).

The immune evasion promoted by SpnA could involve interference with complement function and phagocytosis, which raised the central question of this investigation; could SpnA at the bacterial surface bind and retain secreted IdeS at the surface to cleave opsonizing IgG and prevent phagocytosis? The results demonstrate that IdeS indeed binds to SpnA, and that, in complex with SpnA, IdeS efficiently cleaves IgG antibodies binding to the streptococcal surface.

## Results

### Phagocytosis experiments

The *S. pyogenes* AP1 isolate of the M1 serotype has been studied in our laboratory for more than 30 years. It expresses two surface molecules, protein H and M1 protein, which both bind human IgG antibodies *via* the Fc region (7, 9). In this study, this IgGFc-binding activity was a confounding factor, and an AP1 mutant strain (BMJ71) lacking these proteins (25) was therefore regarded as eminently fit for the present investigation. However, since previous work has shown that IdeS protects AP1 bacteria against phagocytosis (26), we wanted to ensure that this property applies also for the BMJ71 mutant before using the strain throughout the study.

**Figure 1:**
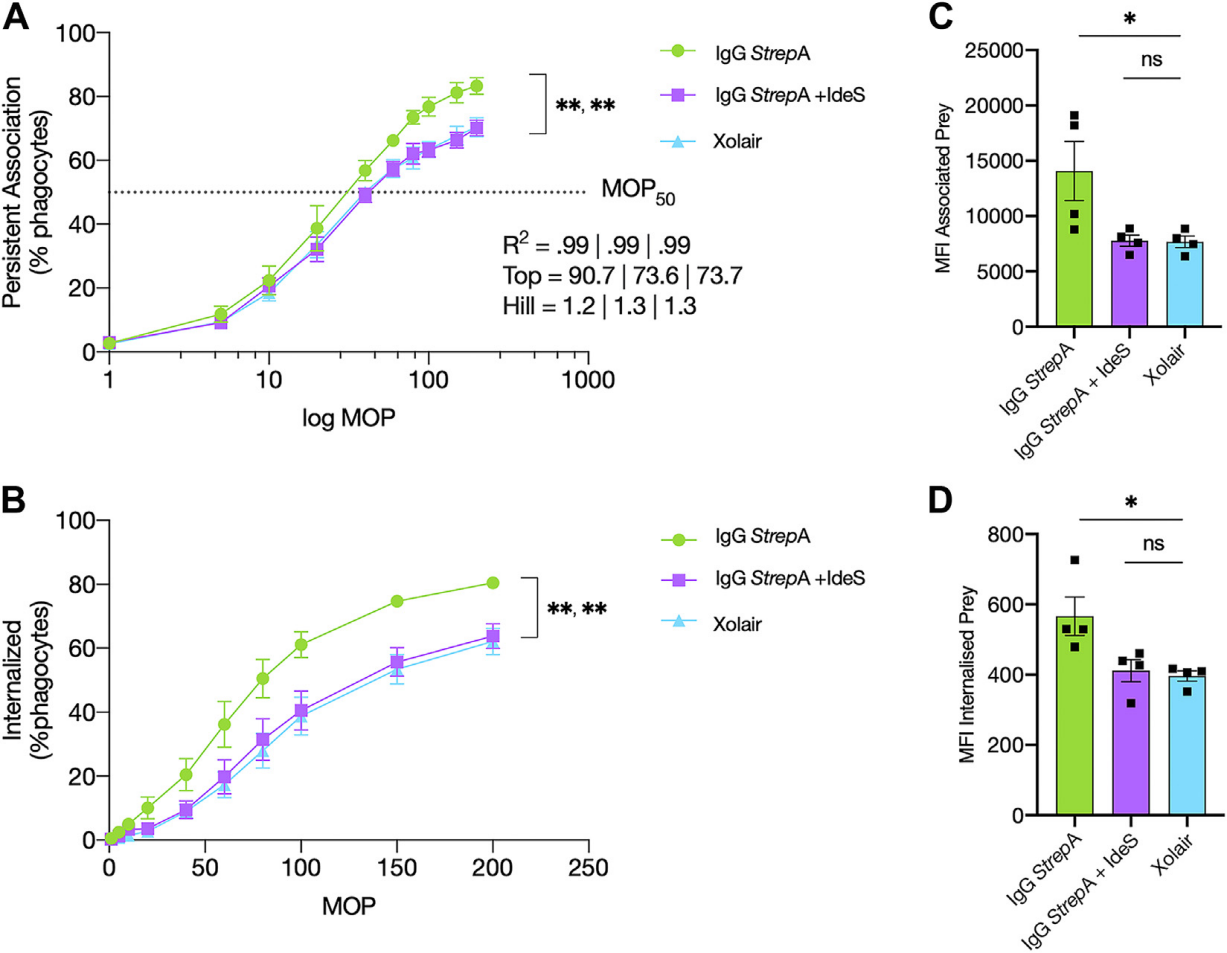
**Phagocytosis experiments.***A*, THP-1 cells were incubated with increasing multiplicities of prey (MOPs) of heat-killed BMJ71 bacteria opsonized with IgG *Strep*A (polyclonal IgG against *Streptococcus pyogenes*), IdeS-cleaved IgG *Strep*A, or Xolair (monoclonal IgG against IgE). The THP-1 cells were allowed to associate with and internalize the bacteria for 30 min before flow cytometric analysis. The curves represent the percentage of cells that were associated with bacteria as a function of the MOP. *R*^2^ indicates that the curves fit a distribution from a population with a normal distribution, and top and Hill coefficient values from the fitted curves are shown. *B*, THP-1 cells were incubated as in (*A*), but only the percentage of cells with internalized bacteria were plotted for each MOP. The data shown in this figure are from the pooled results of four independent experiments. *C*, the mean fluorescence intensity (MFI) of THP-1 cells in the FITC channel indicates the effect of each antibody on bacteria association to THP-1 cells at MOP_50_, which represents the MOP at which there is an equal degree of persistently associated phagocytes. *D*, the MFI of THP-1 cells in the FITC channel indicates the effect of each antibody on bacteria internalization into THP-1 cells at MOP_50_. Error bars represent the SEM. Statistical significance was assessed using one-way ANOVA, and ∗ denotes *p* ≤ 0.05 and ∗∗ denotes *p* ≤ 0.01. IdeS, immunoglobulin G-degrading enzyme of *Streptococcus pyogenes*; IgG, immunoglobulin.

**Supporting Figure S1:**
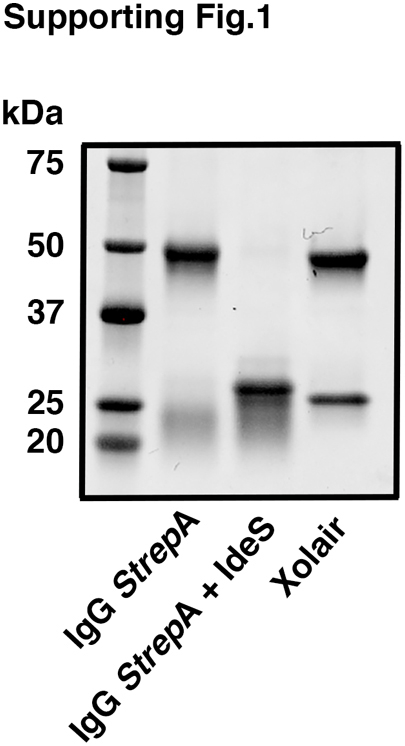
**SDS-PAGE analysis of IgG *Strep*A, IgG *Strep*A cleaved by IdeS, and Xolair.** IgG StrepA (100 μg) was cleaved by IdeS (final concentration of 3 µg/ml) overnight at 37 °C. Intact IgG *StrepA*, cleaved IgG *StrepA*, and Xolair (10 μg) were analyzed by SDS-PAGE (gradient gel 4-20%). The gel was stained with GelCode Blue protein stain.

To assess the effect of IdeS-mediated IgG cleavage on antibody-mediated phagocytosis, we employed persistent association–based normalization (27) where we measure the proportion of phagocytes that are associated with bacteria, and by comparing conditions at similar levels of association, it is possible to normalize for experimental variation. Here, we compared opsonic polyclonal anti-*S. pyogenes* IgG antibodies (IgG *Strep*A) to the same antibodies, which had previously undergone complete cleavage by IdeS. To ascertain the extent of non–Fc receptor-mediated phagocytosis, the activity of these antibodies was compared with a nonbinding antibody control treatment (Xolair, a monoclonal humanized IgG anti-immunoglobulin E antibody). We incubated phagocytic THP-1 cells with pH-sensitive CypHer5-stained bacteria at increasing multiplicities of prey (MOPs). This allowed us to study both the antibodies’ ability to increase phagocyte association as well as internalization. Compared with Xolair, only the intact polyclonal antibody led to an increase in association of BMJ71 bacteria with THP-1 cells, whereas no difference was seen with the IdeS-cleaved IgG *Strep*A antibody (Fig. 1*A*). The same was found regarding internalization where already at lower MOPs the intact antibody led to a larger percentage of THP-1 cells with internalized prey (Fig. 1*B*). This is seen as a left shift in the curve, meaning that fewer bacteria are required to achieve maximal association and internalization. A similar trend was observed when comparing the antibody effects on phagocytosis at equivalent degree of active phagocytes (persistent association, MOP_50_). The intact antibody was significantly more effective at both promoting a larger number of associated bacteria with THP-1 cells (mean fluorescence intensity ± SEM; 14,078.3 ± 2667.7, 7779.7 ± 503.6, and 7674 ± 521.4; Fig. 1*C*) as well as the number of internalized bacteria (mean fluorescence intensity ± SEM; 566.1 ± 54.6, 411.6 ± 31.6, and 396.4 ± 14.8; Fig. 1*D*). The phagocytosis data showed that only intact IgG *Strep*A antibodies (see Fig. S1 for gel) were able to promote efficient phagocytosis of BMJ71 bacteria.

### Analysis of surface-associated SpnA and IdeS

**Figure 2:**
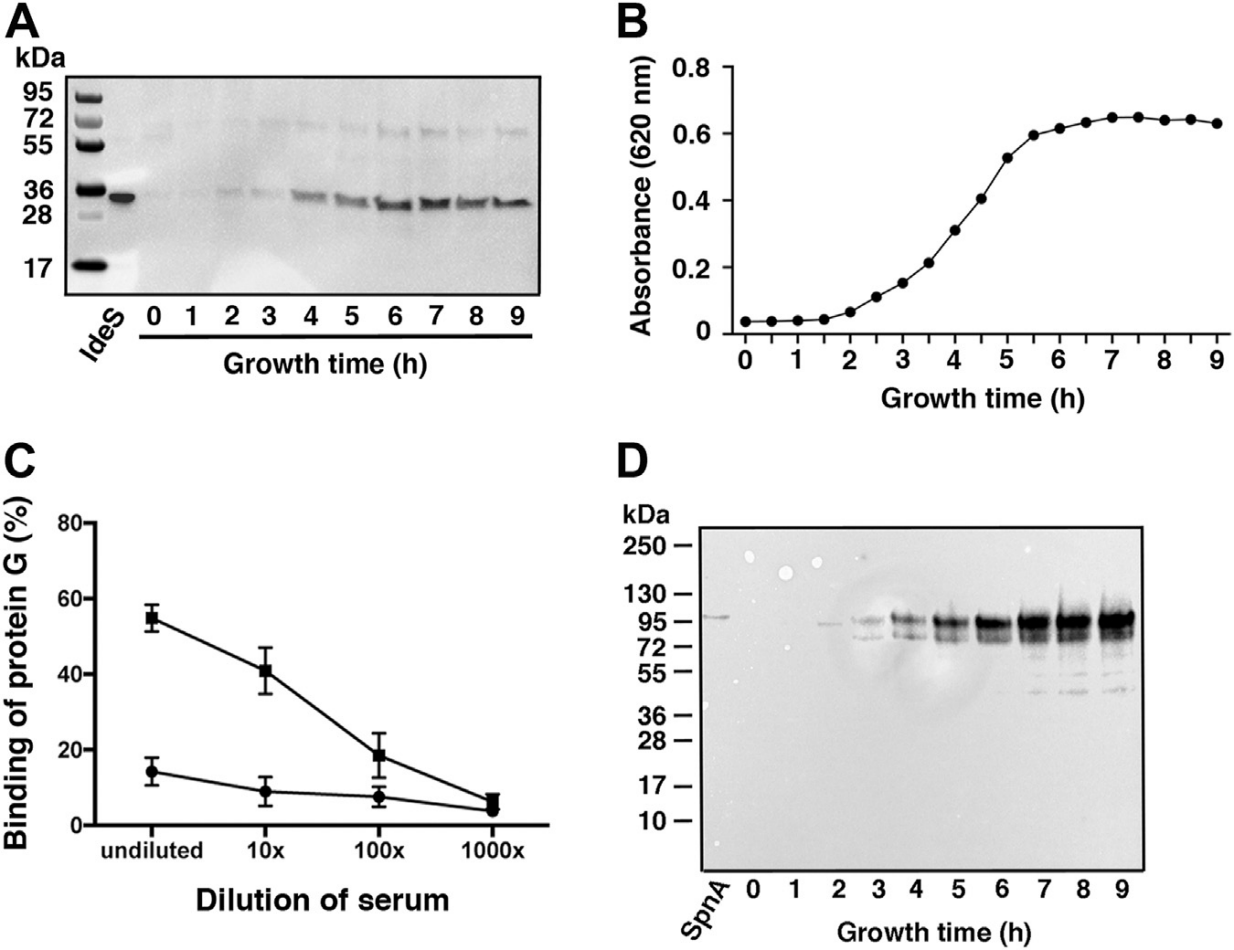
**Analysis of IdeS and SpnA expression.***A*, BMJ71 growth medium from various time points was precipitated with TCA and analyzed by Western blot using antiserum against IdeS (diluted 1:1000) as the probe. Purified IdeS (50 ng) was used as positive control. *B*, the corresponding growth curve of BMJ71 is shown. *C*, BMJ71 bacteria were incubated with indicated dilutions of preimmune SpnA serum (•) or anti-SpnA serum (▪). Binding of IgG antibodies to the bacteria was detected with radiolabeled protein G. Data shown are mean values from three experiments ± SD. *D*, the bacteria from (*B*) were boiled in SDS-PAGE sample buffer and analyzed by Western blot using antiserum against SpnA (diluted 1:1000) as the probe. Purified SpnA (50 ng) was used as positive control. IdeS, immunoglobulin G-degrading enzyme of *Streptococcus pyogenes*; IgG, immunoglobulin G; SpnA, *S*. *pyogenes* nuclease A; TCA, trichloroacetic acid.

**Figure 3:**
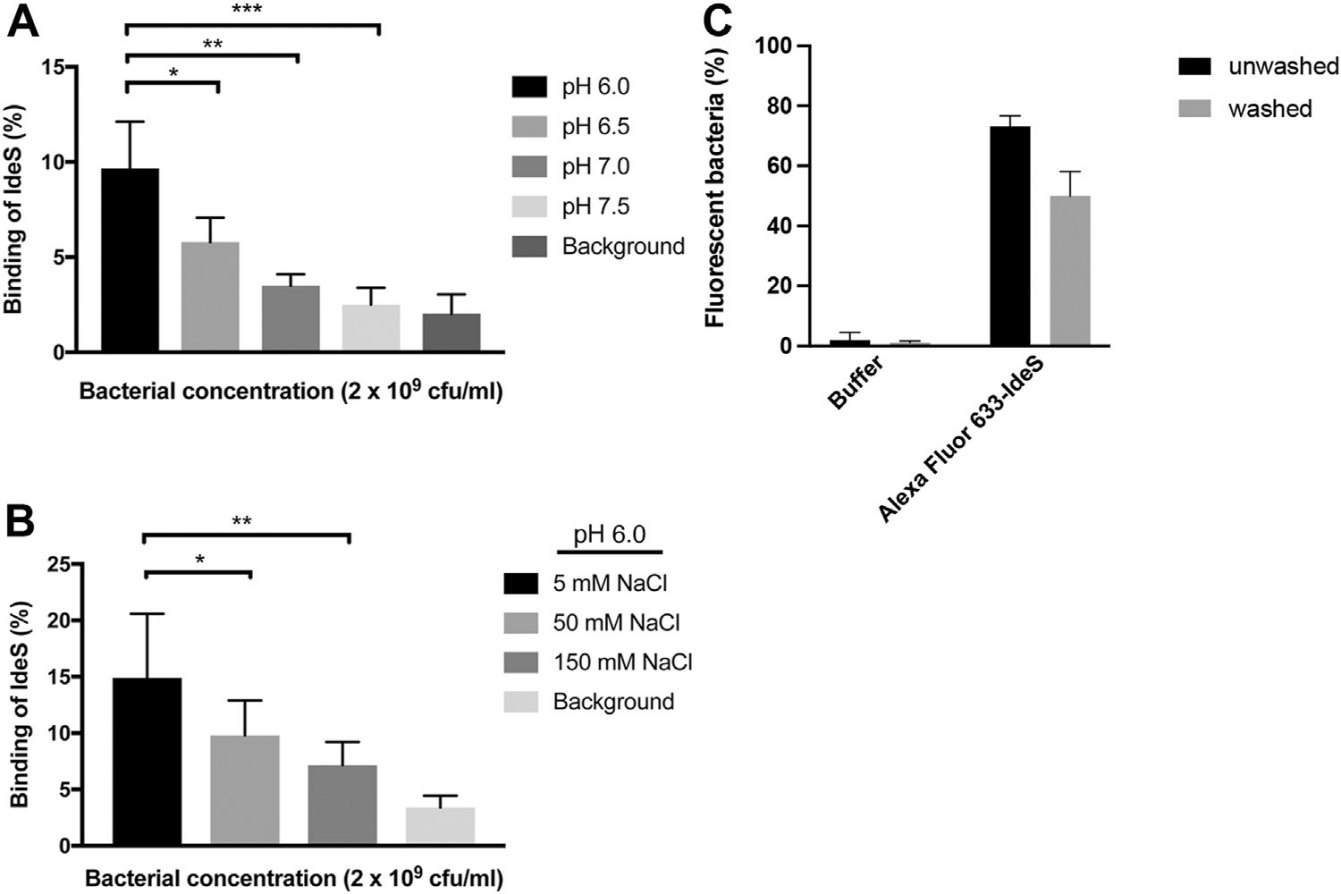
**Binding of IdeS to BMJ71.***A*, BMJ71 bacteria (2 × 10^9^ cfu/ml) were tested for binding of radiolabeled IdeS in 20 mM sodium phosphate buffer + 5 mM NaCl at different pH. Data shown are mean values from three experiments ± SD and were evaluated using one-way ANOVA (pH 6 *versus* pH 6.5, *p* = 0.0226; pH 6 *versus* pH 7, *p* = 0.0011; pH 6 *versus* pH 7.5, *p* = 0.0003). *B*, BMJ71 bacteria (2 × 10^9^ cfu/ml) were tested for binding of radiolabeled IdeS in 20 mM sodium phosphate buffer (pH 6.0) + various concentrations of NaCl. Data shown are mean values from three experiments ± SD and were evaluated using one-way ANOVA (5 mM NaCl *versus* 50 mM NaCl, *p* = 0.0488; 5 mM NaCl *versus* 150 mM NaCl, *p* = 0.0026). *C*, stationary phase BMJ71 bacteria, unwashed or washed (2 × 10^7^ cfu/ml in 20 mM phosphate buffer [pH 6.0] + 5 mM NaCl), were incubated with Alexa Fluor 633-IdeS for 30 min and analyzed by flow cytometry. The percentage of bacterial cells positive for IdeS is shown. For unwashed samples, 10,000 events were recorded, and for washed samples, 3000 events were recorded. Mean values ± SD of three experiments are shown. cfu, colony-forming unit; IdeS, immunoglobulin G-degrading enzyme of *Streptococcus pyogenes*.

All investigated *S. pyogenes* isolates have shown IdeS secretion starting at early exponential growth phase, and this is valid also for the BMJ71 mutant (Fig. 2, *A* and *B*). To verify that SpnA is present at the surface of BMJ71, we incubated the bacteria with dilutions of preimmune or immune serum raised against SpnA. The bacteria were washed, and IgG antibodies bound to the surface were detected with Fc-binding radiolabeled protein G. The enhanced binding of protein G following incubation with immune serum compared with preimmune serum (Fig. 2*C*) demonstrated the presence of SpnA at the bacterial surface, which was further verified by Western blot analysis of boiled bacteria (Fig. 2*D*). SpnA exerts maximum DNase activity at a pH range of 5.5 to 7 (23), and the binding of radiolabeled IdeS to the surface of BMJ71 was tested at various pH as well as at different concentrations of NaCl. The highest binding of IdeS to BMJ71 bacteria was obtained at pH 6.0 (Fig. 3*A*), and when tested at pH 6.0 and different salt concentrations, maximum binding was recorded in a buffer containing 5 mM NaCl (Fig. 3*B*). These conditions, acidic pH and low salt concentration, correspond to what is found in resting saliva (28, 29). At 150 mM NaCl, the salt concentration of human plasma binding was significantly reduced (Fig. 3*B*). The binding of IdeS to the bacterial surface was also analyzed with flow cytometry. BMJ71 bacteria were incubated with Alexa Fluor 633-IdeS, and binding was defined as the percentage of bacteria positive for the ligand as compared with the control. A clear interaction was obtained with both unwashed and washed bacteria (Fig. 3*C*). The experiments described previously demonstrate the presence of SpnA at the surface of BMJ71 bacteria, and that externally added IdeS binds to the bacteria.

**Figure 4:**
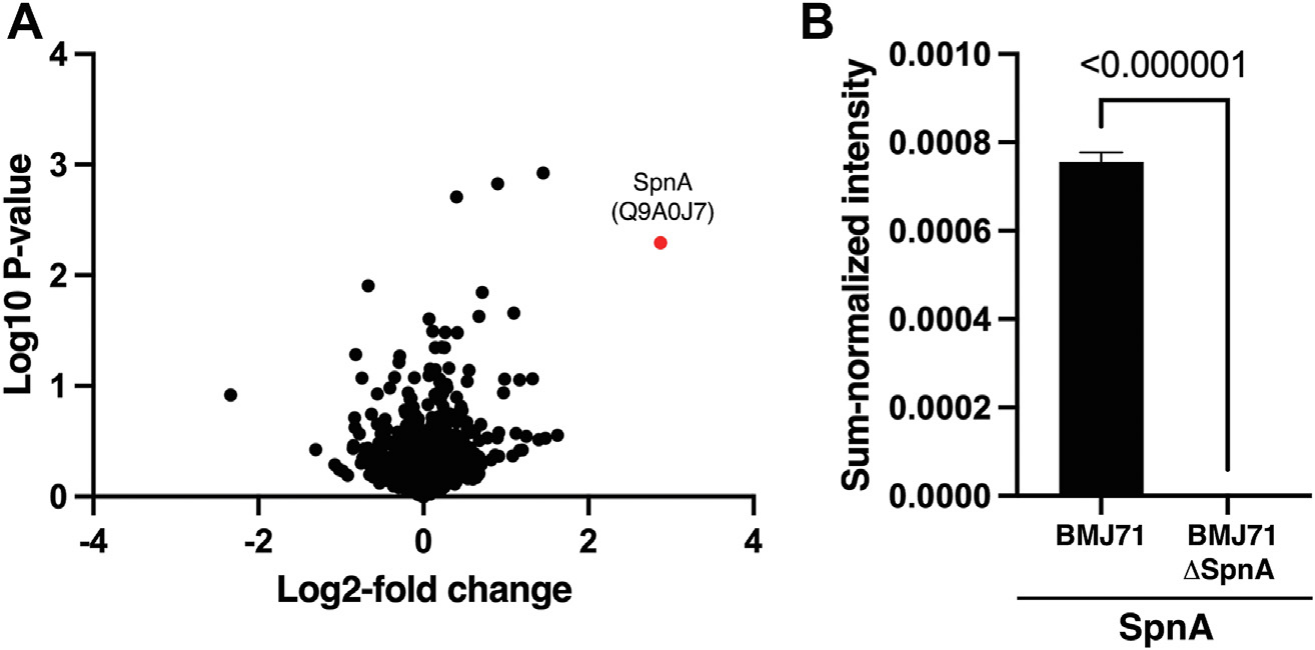
**SpnA mutant; total proteome.** A, volcano plot comparing the total proteome of the BMJ71 and BMJ71 ΔSpnA strains generated in Perseus. The only significant difference is the lack of SpnA (UniProt ID: Q9A0J7) in the BMJ71 ΔSpnA strain, as indicated by a *red sphere* as compared with proteins showing no significant difference between the strains (*black spheres*) (threshold for significance: log2 fold <2 and log10 *p* value <2). *B*, boxplots demonstrating the absence of SpnA in the BMJ71 ΔSpnA mutant. The volcano plots were calculated in Perseus and plotted in GraphPad Prism 9. The boxplots were generated in GraphPad Prism 9. SpnA, *Streptococcus pyogenes* nuclease A.

**Figure 5:**
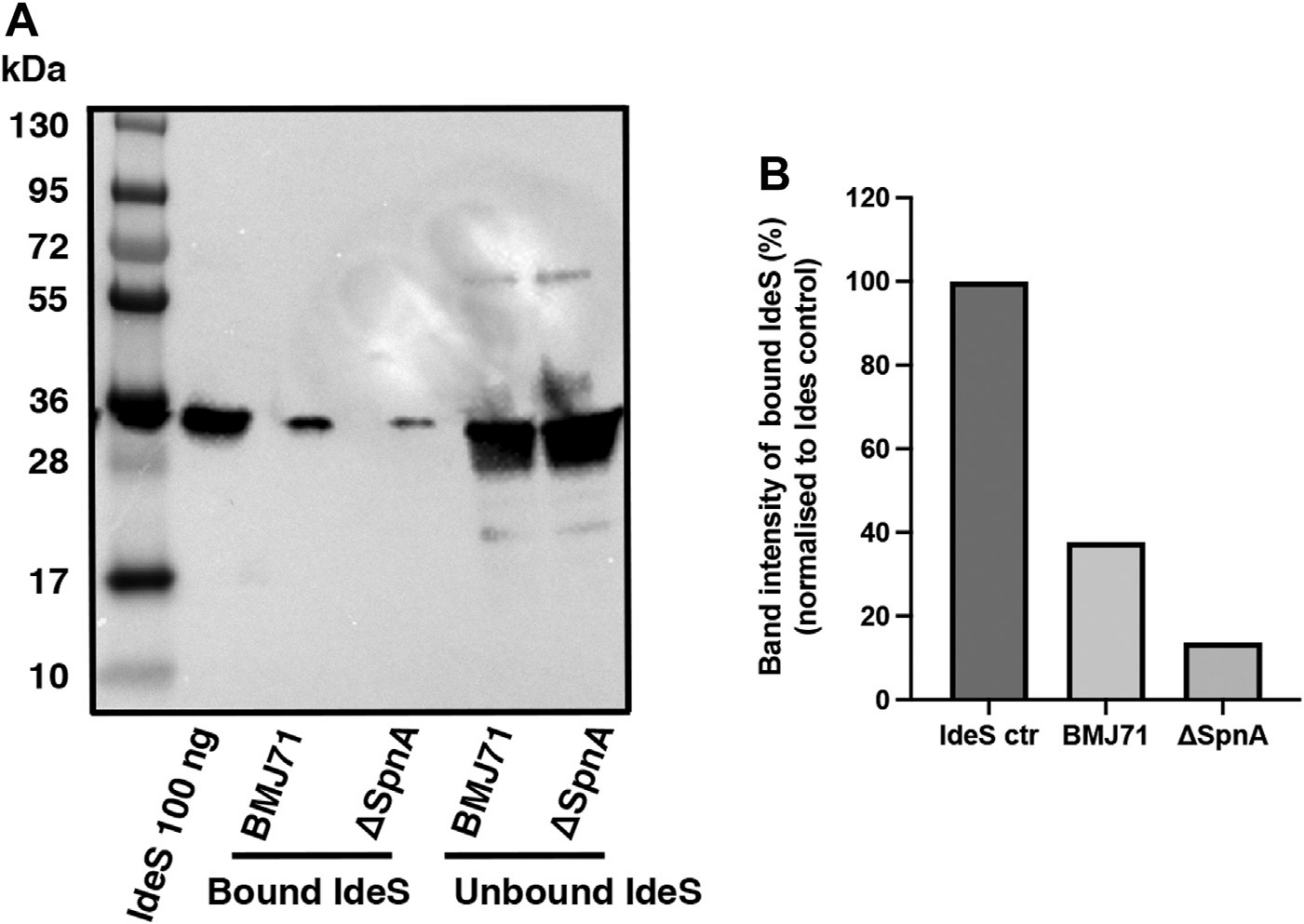
**Binding of IdeS to the bacterial surface.***A*, BMJ71 and BMJ71ΔSpnA were grown to midlogarithmic phase and washed with 20 mM phosphate buffer + 5 mM NaCl, pH 6. One milliliter of bacterial suspension (2 × 10^9^ cfu/ml) was incubated with 50 μg IdeS at 37 °C for 1 h. The supernatants were collected (unbound IdeS), and the bacteria were washed three times with the aforementioned buffer, resuspended in 200 μl 0.1 M glycine–HCl buffer (pH 2), and incubated 30 min at room temperature. Supernatants were collected, pH was raised to 7.0, and precipitated with TCA. Samples were analyzed by Western blot using antiserum against IdeS (diluted 1:1000) as the probe. *B*, band intensity analysis of the Western blot depicted in (*A*) using the Fiji software. The band intensity of bound IdeS was normalized to the band intensity of the IdeS control 100 ng (100%), resulting in 38 ng IdeS bound to the surface of BMJ71 (38%) and 14 ng IdeS bound to BMJ71ΔSpnA (14%). cfu, colony-forming unit; IdeS, immunoglobulin G-degrading enzyme of *Streptococcus pyogenes*; TCA, trichloroacetic acid.

**Supporting Figure S2:**
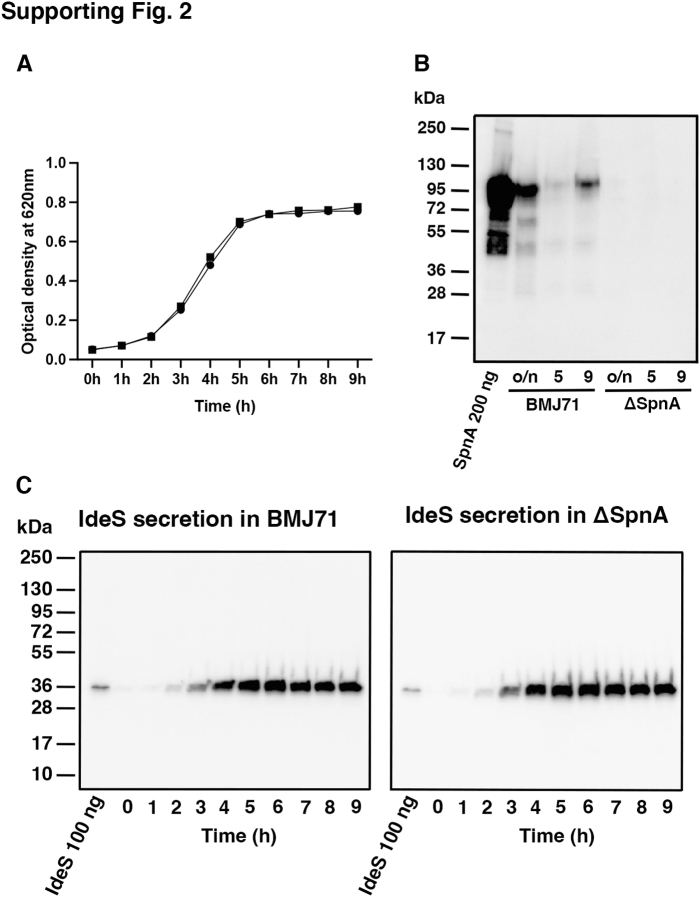
**Analysis of the *SpnA* mutant strain.***A,* BMJ71 and BMJ71ΔSpnA were grown overnight in TH broth. A portion of 400 μl from each strain was transferred to a new tube with 8 ml TH and cultivated for 9 h. The optical density at 620 nm was measured at indicated time points. *B,* BMJ71 and BMJ71ΔSpnA were grown overnight in TH broth. A portion of 400 μl was transferred to two tubes (for each strain) with 8 ml fresh TH and cultivated for 5 h and 9 h. Bacteria from the o/n, 5 h, and 9 h growth were washed with PBS and boiled in SDS-PAGE loading buffer. Samples were analysed by Western blot using antiserum against SpnA (diluted 1:1000) as the probe. *C,* BMJ71 and ΔSpnA were grown overnight in TH broth. A portion of 400 μl was transferred to 10 tubes (for each strain) with 8 ml TH and cultivated for the indicated timepoints. At each timepoint, the supernatants were collected, sterile filtered, and precipitated with TCA. All samples were subjected to Western blot using antiserum against IdeS (diluted 1:1000) as the probe.

To further validate this interaction, a BMJ71 *spnA* mutant was generated. Analyses of bacterial growth showed no difference between strain BMJ71 and the *spnA* mutant (Fig. S2*A*). Western blot of boiled bacteria verified the lack of SpnA at the surface of the mutant strain (Fig. S2*B*), and the expression of IdeS did not differ between the strains (Fig. S2*C*). The protein expression of BMJ71 and the mutant was further assayed by quantitative label-free proteomics mass spectrometry of intact bacteria. Here, of 1169 proteins identified, the only significant difference in the protein expression between the two strains was the lack of SpnA in the *spnA* mutant (Fig. 4, *A* and *B*). The strains were now preincubated with IdeS, washed and incubated with glycine buffer, pH 2.0, to release surface-associated IdeS. This material was subjected to Western blot analysis, which showed less extracellular IdeS associated with the *spnA* mutant compared with the BMJ71 strain; 14 ng *versus* 38 ng (Fig. 5, *A* and *B*). When repeated, the experiment showed similar results; 12 ng *versus* 29 ng. The results identify SpnA as the major IdeS binding surface protein, but the observation that also the *spnA* mutant has IdeS binding activity suggests that additional surface-associated molecules also contribute to IdeS binding. The identification and characterizing of such possible other structures interacting with IdeS will be the subject of future studies.

### Interaction between purified streptococcal nuclease SpnA and IdeS

**Figure 6:**
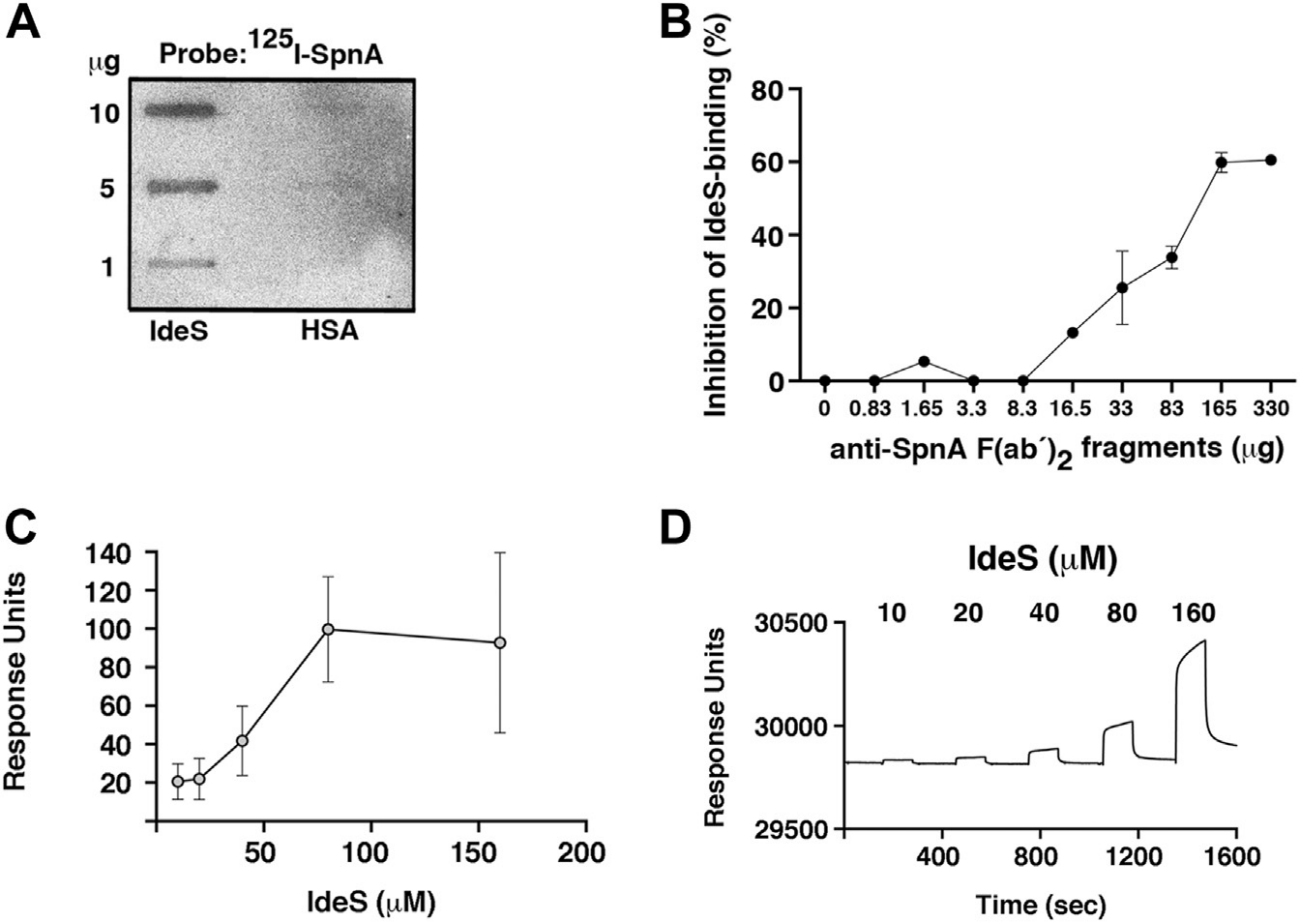
**Interaction between SpnA and IdeS.***A*, indicated amounts of IdeS and HSA were applied in slots to a PVDF filter and probed with radiolabeled SpnA. *B*, the binding of IdeS to BMJ71 bacteria was inhibited with indicated amounts of anti-SpnA F(ab’)_2_ fragments. Data from two independent experiments are shown. *C* and *D*, surface plasmon resonance spectroscopy of the interaction between SpnA and IdeS. *C*, SpnA was immobilized on a CM5 chip, and IdeS was injected at different concentrations. Steady state of three experiments is shown. *D*, a representative experiment is depicted, and the *K*_*d*_ of the interaction between SpnA and IdeS was determined to be 5.33 × 10^−5^ M. Fab, fragment antigen-binding; HSA, human serum albumin; IdeS, immunoglobulin G-degrading enzyme of *Streptococcus pyogenes;* PVDF, polyvinylidene difluoride; SpnA, *Streptococcus pyogenes* nuclease A.

Ever since IdeS was discovered as a uniquely specific IgG-cleaving proteinase (13), we have searched for a mechanism that could withhold at least a portion of the secreted IdeS at the bacterial surface, where it could degrade opsonizing IgG antibodies. The antibactericidal properties reported for SpnA (23) presented the possibility that the nuclease also could have IdeS-binding activity. Therefore, a direct interaction between the two proteins needed to be demonstrated to further prove the binding. Thus, purified IdeS and human serum albumin were applied to a polyvinylidene difluoride membrane and probed with radiolabeled SpnA, and a dose-dependent binding of SpnA was obtained to IdeS but not to the negative control human serum albumin (Fig. 6*A*). The binding of radiolabeled IdeS to BMJ71 bacteria could also be inhibited with anti-SpnA F(ab’)_2_ fragments demonstrating an interaction of IdeS with SpnA at the bacterial surface (Fig. 6*B*). However, the inhibition is not complete (around 60%), again suggesting that other surface structures could also bind IdeS. This agrees well with the data of Figure 5 showing that the *spnA* mutant, although lower than the BMJ71 strain, still has some IdeS binding activity. Finally, plasmon surface spectroscopy experiments showed a dose-dependent binding of IdeS to immobilized SpnA with a *K*_*d*_ of 5.33 × 10^−5^ M (Fig. 6, *C* and *D*). Taken together, the results demonstrate binding between IdeS and SpnA and further support an interaction between IdeS and SpnA at the bacterial surface.

### SpnA and IdeS form IgG-cleaving complexes

**Figure 7:**
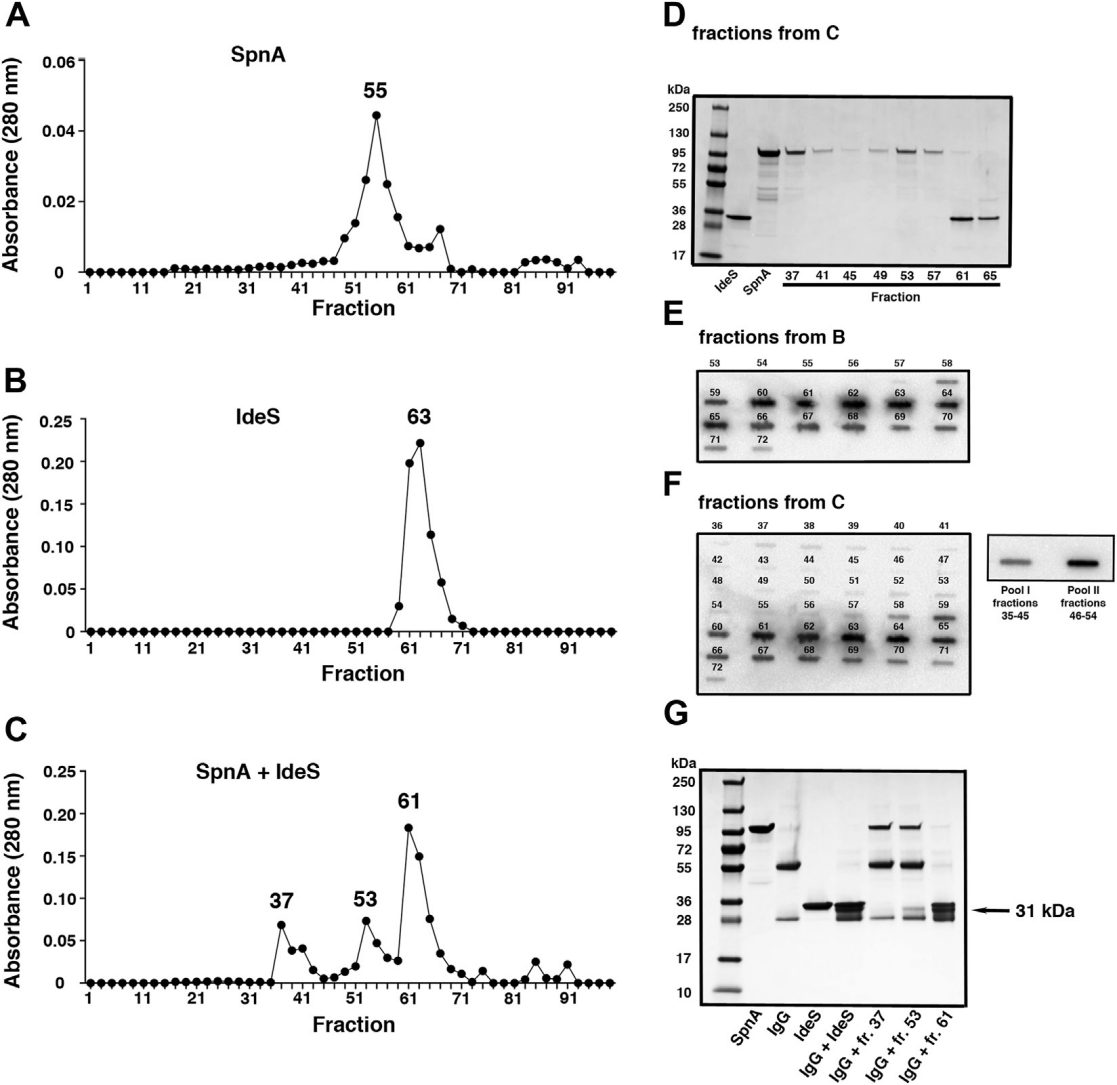
**Formation of IdeS–SpnA complexes that cleave IgG antibodies.** SpnA and/or IdeS were separated on a Superose 12 gel filtration column in 20 mM phosphate buffer (pH 6.0) + 5 mM NaCl. Fractions of 0.25 ml were collected, and the absorbance at 280 nm was measured. *A*, chromatogram of SpnA. *B*, chromatogram of IdeS. *C*, chromatogram of SpnA and IdeS preincubated together for 30 min at room temperature before gel filtration. *D*, SDS-PAGE analysis of 10 μl fractions from (*C*). *E* and *F*, fractions from (*B* and *C*) (100 μl) and pooled fractions from (*C*) (pool I and pool II) were applied in slots to PVDF membranes, and the membranes were probed with anti-IdeS followed by HRP-conjugated protein G. *G*, fractions 37, 53, and 61 (10 μl) from the gel filtration of SpnA + IdeS (shown in *C*) were incubated with IgG (3 μg) for 1 h at 37 °C. Samples were analyzed by SDS-PAGE. Lane 1: SpnA 3 μg; lane 2: IgG 3 μg; lane 3: IdeS 2 μg; lane 4: IgG 3 μg + IdeS 2 μg; lane 5: IgG 3 μg + fraction 37 (10 μl); lane 6: IgG 3 μg + fraction 53 (10 μl); and lane 7: IgG 3 μg + fraction 61 (10 μl). HRP, horseradish peroxidase; IdeS, immunoglobulin G-degrading enzyme of *Streptococcus pyogenes*; IgG, immunoglobulin G; PVDF, polyvinylidene difluoride; SpnA, *Streptococcus pyogenes* nuclease A.

**Supporting Figure S3:**
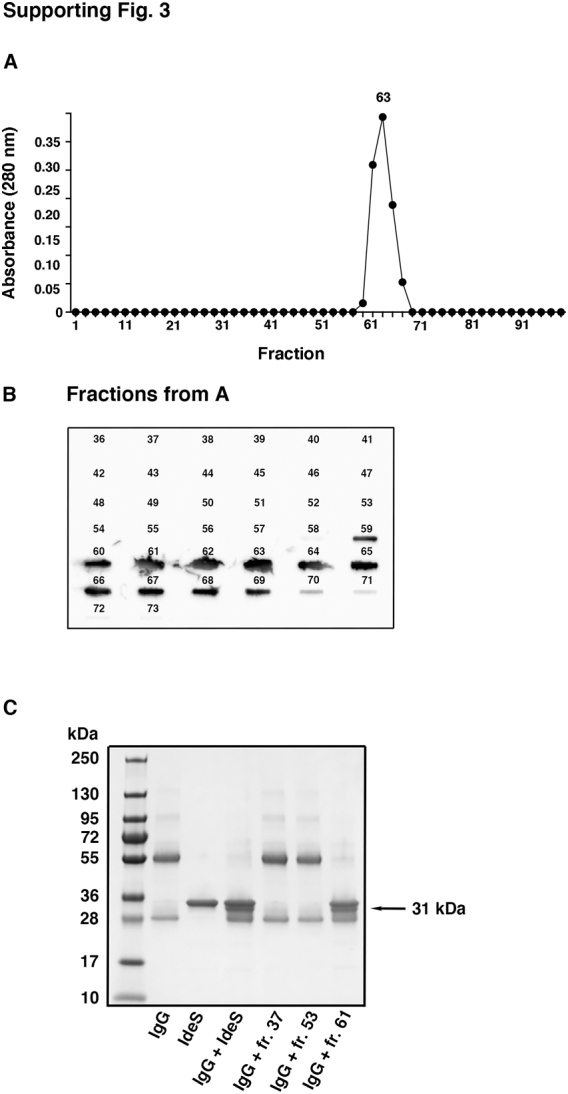
**Analysis of IdeS in gel filtration experiments.***A,* IdeS was separated on a Superose 12 gel filtration column in 20 mM phosphate buffer pH 6.0 + 5 mM NaCl. Fractions of 0.25 ml were collected and the absorbance at 280 nm was measured. *B,* ten microliters of the fractions from (*A*) were applied in slots to a PVDF membrane, probed with anti-IdeS antibodies followed by HRP-conjugated IgGFc-binding protein G. *C,* SDS-PAGE of purified IgG, IdeS, IgG+IdeS, and IgG+fractions 37, 53, or 61 from the chromatogram of (*A*). The 31 kDa fragment of IgG heavy chains generated by IdeS cleavage is indicated by the *arrow*.

To investigate whether SpnA and IdeS form complexes where IdeS still has IgG-cleaving activity, gel filtration experiments were conducted. First, SpnA and IdeS samples were separately run on a gel filtration Superose 12 column, and the resulting chromatograms show major peaks in fractions 55 and 63, respectively (Fig. 7, *A* and *B*). Additional information to Figure 7*B* is shown in Fig. S3; no IdeS- or IgG-cleaving activity was observed in fractions outside the peak fractions of Figure 7*B*. An equimolar mixture of SpnA and IdeS was then incubated for 30 min at room temperature and subjected to the Superose 12 column. The resulting chromatogram (Fig. 7*C*) shows two new peaks at fractions 53 and 37, which are absent in the SpnA and IdeS chromatograms. Fractions from the separation of IdeS–SpnA (Fig. 7*C*) were analyzed by SDS-PAGE under reducing conditions. Figure 7*D* shows that SpnA is present not only in fractions 49 to 57 but also in fraction 37 (this fraction corresponds to the void volume of the column determined by separation of blue dextran), showing that the protein forms larger complexes following incubation with IdeS. A band corresponding to the size of IdeS was seen in fractions 61 and 65 (Fig. 7*D*). To increase the sensitivity of IdeS detection, fractions were subjected to slot binding assays using antibodies against IdeS as the probe. Figure 7*E* shows fractions of the IdeS peak in the chromatogram of Figure 7*B*, whereas analysis of IdeS–SpnA fractions (chromatogram in Fig. 7*C*) identified IdeS also in fractions 56 and 57. In addition, when fractions 35 to 45 and fractions 46 to 54 were pooled and concentrated (pools I and II, respectively), IdeS was detected also in this material (Fig. 7*F*). The wide distribution of small amounts of IdeS over earlier-running fractions may indicate association with SpnA complexes of different sizes. Fractions 37, 53, and 61 from the separation of IdeS–SpnA (Fig. 7*C*) were then incubated with human IgG at 37 °C for 1 h, and cleavage was analyzed by SDS-PAGE. As anticipated, IgG cleavage was complete in fraction 61, a peak fraction also in the chromatogram of Figure 7*B* where IdeS was run alone. However, partial IgG cleavage was also obtained with fraction 53, demonstrating the presence of proteolytically active IdeS in this fraction (Fig. 7*G*). No cleavage of IgG was obtained in fraction 37 (Fig. 7*G*), probably because of the low concentration of IdeS (Fig. 7*F*). In summary, the results show that IdeS forms complexes with SpnA, and that IdeS in these complexes has IgG-cleaving activity.

### Super-resolution microscopy shows colocalization of SpnA and IdeS at the bacterial surface

**Figure 8:**
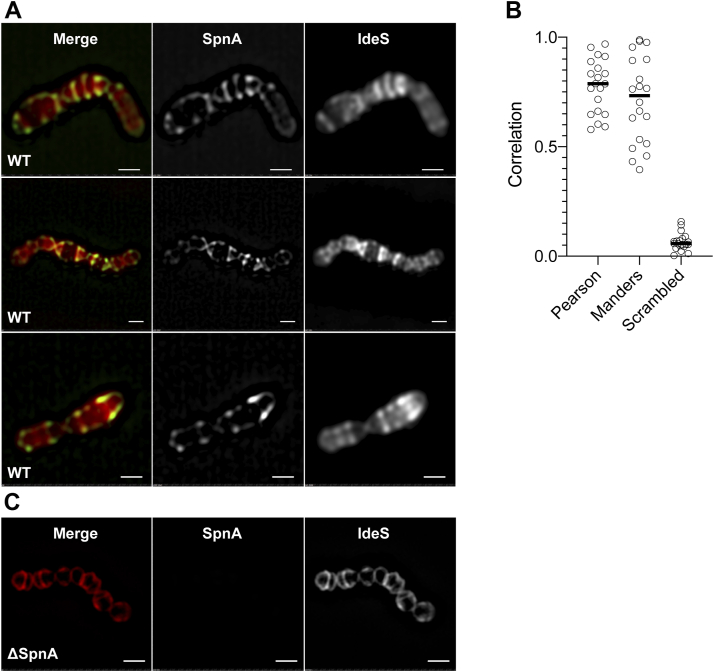
**Colocalization of SpnA and IdeS at the bacterial surface.***A*, SIM images of SpnA and IdeS at the bacterial surface of *Streptococcus pyogenes strain* BMJ71. Three representative image series are shown with single-channel images (Alexa 647-IdeS and Alexa 488-labeled anti-SpnA antibodies) in grayscale and merged channels in color. *Green* and *red* represent SpnA and IdeS, respectively, and the corresponding monochromatic images (as they are collected) are shown. In the merged image, *yellow* appears when there is a strong signal in both the *green* and the *red channel*. N = 20 images. Scale bar represents 1 μm. *B*, colocalization analysis of images from (*A*). Pearson's correlation analysis resulted in mean correlation of 0.77 ± 0.12 SD, Mander's mean correlation was 0.71 ± 0.20 SD, and the scrambled control data had a correlation of 0.07 ± 0.04 SD. N = 20 images. *C*, SIM images of BMJ71ΔSpnA as performed in (*A*). As the SpnA signal, as expected, was too weak, no correlation analysis was performed. Scale bar represents 1 μm. IdeS, immunoglobulin G-degrading enzyme of *Streptococcus pyogenes*; SIM, structured illumination microscopy; SpnA, *S*. *pyogenes* nuclease A.

Having established that SpnA and IdeS form complexes in solution, we next wanted to investigate whether this also occurs at the surface of live bacteria. BMJ71 bacteria were grown to exponential phase and then incubated with Alexa Fluor 647-IdeS and Alexa Fluor 488-anti-SpnA F(ab’)_2_ fragments, followed by fixation of the bacteria. Super-resolution 3D structured illumination microscopy (SIM) was then used to analyze the localization of the two proteins at the bacterial surface. The images show that in most cases the IdeS signal localizes well to regions where SpnA is located (Fig. 8*A*). There is a degree of variability in the patterns of both SpnA and IdeS, likely because of variations in the expression of SpnA at the surface of individual bacteria. However, a weak binding of IdeS across a large portion of the bacterial surface is also observed. Together with other results described previously, this further indicates that SpnA is the major, but not the only, surface structure binding IdeS. Quantitative analysis of correlation resulted in a strong positive correlation between IdeS and SpnA using either Pearson's or Mander's correlation analysis and with both methods significantly higher than scrambled pixels of the same images (Fig. 8*B*). Overall, the super-resolution data show that IdeS and SpnA colocalize at the surface of live bacteria. Finally, the SIM image of Figure 8*C* shows fewer foci at the surface of *spnA* mutant bacteria and less of the diffuse internal signal observed with BMJ71 bacteria.

### IdeS at the bacterial surface cleaves IgG

**Figure 9:**
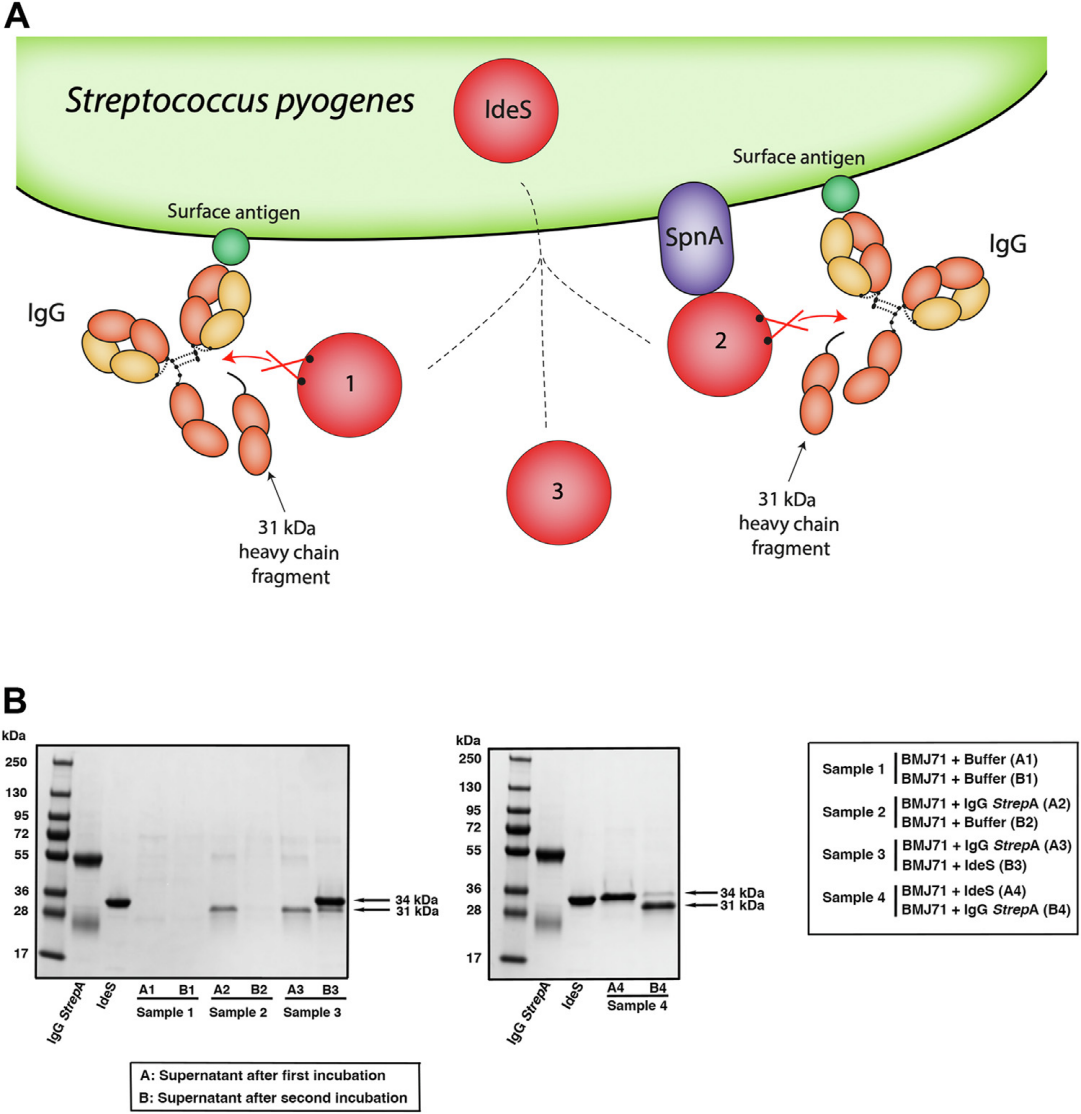
**IdeS is present at the bacterial surface where it cleaves IgG.***A*, schematic representation of IgG, IdeS, and SpnA at the surface of *Streptococcus pyogenes*. IgG antibodies bound to *S. pyogenes* surface antigens are cleaved either directly by secreted IdeS (1) or by IdeS primarily bound to SpnA (2). This generates a 31 kDa fragment of the heavy chain, whereas the F(ab')_2_ fragment remains bound to the bacterial surface. Secreted IdeS may also cleave IgG antibodies at a distance from the bacteria (3). *B*, intact IgG (*second lane* from the *left*) run on SDS-PAGE under reducing conditions (breaking the disulphide bridges between the chains) gives rise to bands of 25 kDa (light chains) and 56 kDa (heavy chains), whereas IdeS migrates as a 34 kDa band (*third lane* from the *left*). Four samples of BMJ71 bacteria at midlogarithmic phase were incubated with buffer, IgG *Strep*A (IgG antibodies specific for *S. pyogenes*), IgG *strep*A, or IdeS for 1 h at room temperature. Bacteria were pelleted, and supernatants (A1–A4) were collected and precipitated with TCA. The saved pellets were washed, resuspended, and incubated in buffer, IdeS, or IgG *Strep*A. Bacteria were spun down, and the four supernatants after this second incubation (B1–B4) were also precipitated with TCA. Finally, the samples from the eight supernatants were analyzed by SDS-PAGE. *Arrows* indicate IdeS (34 kDa) and the 31 kDa IdeS-generated fragments of IgG heavy chains (Fig. 9*A*). The control sample 1 (supernatants A1 and B1) shows that no proteins were released from the bacterial surface during incubation in buffer only. Fab, fragment antigen-binding; IdeS, immunoglobulin G-degrading enzyme of *Streptococcus pyogenes*; IgG, immunoglobulin G; SpnA, *S. pyogenes* nuclease A; TCA, trichloroacetic acid.

IdeS is a secreted enzyme cleaving IgG and releasing a 31 kDa heavy chain fragment. The enzyme is known to degrade IgG in solution, and when externally added, it also cleaves IgG antibodies bound to cell surfaces. The results described previously showing that secreted IdeS itself binds to *S. pyogenes via* SpnA indicated that the bacteria could be constantly equipped with a pool of IdeS at the bacterial surface ready to cleave IgG antibodies binding to surface-exposed epitopes (Fig. 9*A*).

To challenge this hypothesis, we utilized purified IgG antibodies (IgG *Strep*A) against the C carbohydrate of *S. pyogenes*, which is contained in the cell wall and forms the basis of serological grouping of streptococci (Lancefield groups A–O), where *S. pyogenes* is equivalent to group A streptococci. Two identical samples of washed midlogarithmic BMJ71 bacteria were incubated with these IgG *Strep*A antibodies for 1 h. The bacteria were spun down, and the supernatants were analyzed by SDS-PAGE. As shown in Figure 9*B*, lanes A2 and A3, the added IgG was cleaved; the band corresponding to intact IgG heavy chains is very faint, and the band around 25 kDa, corresponding to the light chains, is barely visible. In contrast, a clear 31 kDa heavy chain fragment has been generated. These results show that most of the added IgG *Strep*A antibodies have bound to the bacterial surface. Here, they are cleaved by IdeS and the 31 kDa fragments are released, whereas the remaining F(ab')_2_ fragments are still bound to their surface antigen (Fig. 9*A*). A small fraction of unbound IgG *Strep*A antibodies is responsible for the faint heavy and light chain bands. The outcome of this experiment demonstrates that IdeS is present at the surface where it efficiently cleaves IgG antibodies. The pelleted bacteria (see previous) were resuspended, washed, and incubated with IdeS resulting in cleavage of the remaining bacteria-bound IgG (Fig. 9*B*, lane B3). In parallel, BMJ71 bacteria were incubated with IdeS for 1 h, and SDS-PAGE analysis showed that most of the IdeS (34 kDa band) was in the supernatant (Fig. 9*B*, lane A4). The pellet was now resuspended, bacteria were washed, and incubated with IgG *Strep*A antibodies for 1 h, resulting in efficient cleavage of all added IgG (Fig. 9*B*, lane B4). This experiment shows that externally added IdeS is bound to the surface where it cleaves IgG antibodies releasing the 31 kDa heavy chain fragment. In addition, the 34 kDa IdeS band in the supernatant demonstrates that IdeS is detached from the surface after dismantling the surface-associated IgG antibodies (Fig. 9*B*, lane B4). As a control, bacteria were incubated in buffer, showing that no proteins were released from the bacterial surface during the incubation times. (Fig. 9*B*, lanes A1 and B1).

**Supporting Figure S4:**
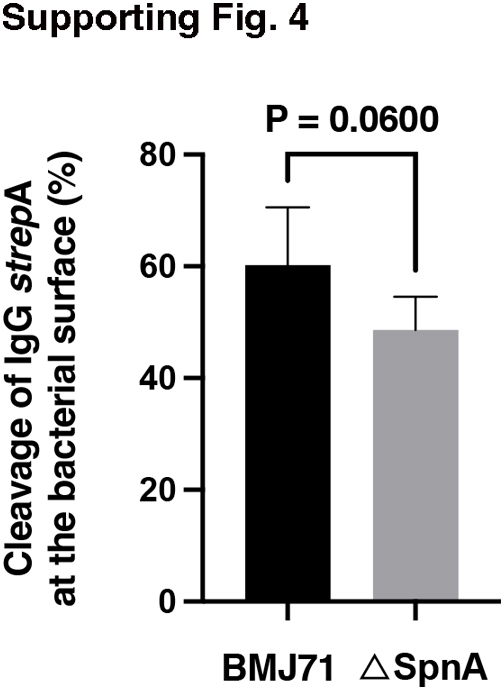
**Cleavage of IgG strepA on the surface of BMJ71 and the *spnA* mutant.** The BMJ71 and the *spnA* mutant were grown overnight in TH. From each culture, 400 μl was transferred to new tubes with 8 ml TH and the cultures were grown to mid-logarithmic phase. The bacteria were washed with 20 mM phosphate buffer pH 6.0 + 5 mM NaCl and resuspended to 1% solution. One milliliter of bacterial solution from each strain was incubated with rabbit anti-strepA IgG (50 μg) for 1 h under rotation at room temperature. The supernatants were collected, sterile filtered, and analyzed with Western blot. Membranes were blocked in 5% dry milk at room temperature, incubated with HRP-conjugated goat anti-rabbit IgG (1:3000) in PBST for 1 h at room temperature, washed with PBST, and developed with chemiluminescence. The intensity of the 56 kDa band (intact IgG heavy chains) and the 31 kDa IdeS-generated fragment was analyzed separately with the Fiji software. These numbers (%) were then combined as total intensity and then the amount of the cleaved 31 kDa band in percent of total intensity was calculated. Data shown are mean values from five experiments ± SD and were evaluated using unpaired T-test.

Additional experiments were performed with BMJ71 and the *spnA* mutant to investigate if there is a difference in the cleavage of IgG antibodies binding to the two strains. In these experiments, externally added IdeS was not employed. The low amount of IdeS occurring at the surface and the presence of surface structures other than SpnA-binding IdeS complicate the experiments that however showed a higher but not significant degree (*p* = 0.0600) of cleavage by the BMJ71 strain (Fig. S4).

## Discussion

All strains of *S. pyogenes* express M protein (for reviews, see Refs. (20, 30)), and based on sequence variations in the NH_2_-terminal tip of these fibrous surface proteins, *S. pyogenes* isolates are grouped into more than 200 M serotypes. Many M serotypes have IgGFc-binding M and M-like proteins, which by binding and blocking Fc protect the bacteria against phagocytic killing. However, this protection only works in an environment with a low concentration of high-affinity Fab-binding IgG antibodies against *S. pyogenes* surface antigens (31). In relation to this study, it is notable that IdeS primarily cleaves these Fab-bound IgG antibodies (32), and to focus the studies on IdeS and high-affinity IgG antibodies, we therefore decided to use the M1 mutant strain BMJ71, which lacks IgGFc-binding M1 protein and protein H. The elimination of nonimmune IgG-binding to the bacteria made it possible to refine the experimental approach and greatly facilitated the interpretation of obtained data.

IdeS is a proteinase with a remarkable specificity; apart from IgG, no other substrate has been identified. The enzyme also cleaves all four human IgG subclasses in the lower hinge region extremely rapidly and efficiently (13); in minutes, one molecule of IdeS cleaves more than 2000 IgG antibodies (33). For cleavage to occur, IdeS first has to bind to IgGFc, and the unique specificity is explained by the requirement for this initial protein–protein interaction (17). The degradation of IgG takes place in two steps where the first heavy chain is cleaved much faster than the second (34, 35, 36). Thus, single-cleaved IgGs are primarily generated before the second Fc half is removed to form F(ab')_2_ fragments. From a functional point of view, this is important since single-cleaved IgG and F(ab')_2_ fragments will retain full antigen-binding activity in solution and when bound to a cell surface antigen, whereas already a single heavy chain cleavage compromises the effector functions of IgG (35). The properties of IdeS described previously indicated that the proteinase could be used as a drug in autoimmune diseases and transplant rejection where IgG antibodies play a pathogenic role, and when IdeS was tested in several animal models of autoimmune disease, this hypothesis was verified (for reviews, see Ref. (33)). In addition, a clinical phase I study on healthy human volunteers showed that a single dose of IdeS within minutes cleaved the entire pool of extracellular IgG (IgG levels were back to normal 2–3 weeks later) without any significant adverse effects (33), results that further underlined the clinical potential of the enzyme. Patients on the waiting list for a kidney transplantation having preformed IgG antibodies against human leukocyte antigens are unlikely to receive a new kidney because of the risk of acute rejection. However, treatment with IdeS eliminated these antibodies and permitted successful transplantation in 24 of 25 such patients (37), and the effect of IdeS in other IgG-driven diseases is currently under investigation.

*S. pyogenes* is one of the most significant bacterial pathogens in the human population responsible for more than 0.5 million deaths and causing at least 700 million cases of pharyngitis and skin infections annually (38). The demonstration that IdeS, a proteinase that has evolved to protect the bacterium against antibody attack, is now being used as a drug in humans was an unexpected finding, but it does of course not shed light on how IdeS is operating at the bacterial surface to dismantle IgG. All investigated *S. pyogenes* isolates express IdeS, and this also applies for the *S. pyogenes* DNase SpnA, implying essential bacterial functions for the two proteins. SpnA has a cell wall–anchoring LPKTG motif and is the only surface-associated DNase, thus far identified in *S. pyogenes* (19, 23). These previous studies also established SpnA as a virulence factor protecting the bacteria against phagocytic killing; an effect that was suggested to be, at least partly, because of degradation of the DNA framework of neutrophil extracellular traps, structures released by activated neutrophils, which entrap and, in some cases, kill bacteria, including *S. pyogenes* (39). IdeS on the other hand, when added to human blood containing opsonizing IgG antibodies against *S. pyogenes*, also protects the bacteria against phagocytic killing (26), indicating that SpnA and IdeS could exert this function mutually.

In our mind, it seemed logical that at least some of a secreted enzyme targeting IgG antibodies bound to the streptococcal surface would be present at this site rather than being entirely discharged into the environment. This assumption was the starting point for the present investigation and raised several questions; does externally added IdeS bind to the bacteria and/or is a fraction of the enzyme continuously present at the surface. If so, to what structure does it bind and is the enzyme proteolytically active when associated with this structure? The results of this study answer these questions, showing that IdeS when added to *S. pyogenes* binds to SpnA at the bacterial surface. Also, without external addition, IdeS is still present at the surface of growing bacteria in complex with SpnA, where the proteinase efficiently cleaves IgG antibodies. The affinity between IdeS and SpnA determined here (*K*_*d*_ = 5.33 × 10^−5^ M) is 10 to 100 times lower compared with what has been reported for the interaction between IdeS and IgG (17, 36, 40, 41). This implies that IdeS will dissociate from the complex with SpnA and bind to and cleave IgG antibodies when they appear at the bacterial surface. It is noteworthy that the results here show that the BMJ71 SpnA mutant still has some, although significantly less, IdeS binding activity compared with the BMJ71 strain (Fig. 5), and that (Fab`)_2_ fragments against SpnA inhibit IdeS binding to BMJ71 bacteria but not completely (Fig. 6*B*). In addition, super-resolution microscopy (SIM) indicated that apart from the major binding of IdeS to the BMJ71 surface *via* SpnA, a weak binding occurred also to other structures (Fig. 8*A*). Together, these different findings suggest that surface structures apart from SpnA contribute to the binding of IdeS to the streptococcal surface. It will be an interesting future task to identify and characterize these structures.

As mentioned previously, cleavage of the first heavy chain is much faster than the second, whereby single-cleaved IgGs are primarily generated. These antibodies will still be bound to their antigen and block the binding of other antibodies to the same epitope and by steric hindrance presumably also block antibodies binding to other closely located antigenic epitopes, whereas the removal of one Fc half (corresponding to the 31 kDa heavy chain fragment shown in Fig. 9*A*) is enough to prevent Fc receptor interactions (35). Moreover, previous work has shown that Fc halves generated by IdeS cleavage prime neutrophils (42). Fc halves will be released from IgG cleaved both at the bacterial surface and from IgG degraded by IdeS in solution, and as a result, neutrophils are primed at a distance from the infection site. Primed neutrophils are easily activated by, for example, immune complexes, and their activation and discharge of granular content will induce an inflammatory reaction, a hallmark of *S. pyogenes* infection. IdeS also hydrolyzes IgG being part of the B-cell receptor, thereby interfering with antigen binding to the receptor (43). This will temporarily silence memory B-cells and prevent their response to antigenic stimulation and transition into antibody-producing cells, another immunosuppressive effect of IdeS.

SpnA has maximum DNAse activity at acidic pH indicating that the protein adapts a functionally favorable configuration at this pH. Acidic pH and low salt concentration are also characteristics for the habitat of *S. pyogenes.* From an evolutionary and functional point of view, it is therefore noteworthy that the interaction between SpnA and IdeS also has a maximum at low pH and salt concentration.

To summarize, this study shows that IdeS, a proteinase secreted by a major human pathogen, associates with a DNase at the bacterial surface, where it subsequently cleaves IgG antibodies binding to the surface. The highly specific and efficient cleavage of IgG by IdeS, both immobilized at cell surfaces and in solution, results in the interference with several host mechanisms important for *S. pyogenes* clearance. The results underline the complexity of the molecular interplay between a bacterium and its human host and provide an example of how evolution may create a protein with multiple effects adding selective advantages to a colonizing microbe.

## Experimental procedures

### Bacteria and growth conditions

The *S. pyogenes* AP1 mutant strain BMJ71 has been described previously (25). The deletion mutant BMJ71ΔSpnA is described later. Bacteria were cultivated in Todd–Hewitt (TH) broth (Difco) at 37 °C, 5% CO_2,_ and for cultivation of BMJ71, tetracycline was added to a final concentration of 5 μg/ml. For total proteome analysis, BMJ71 and BMJ71ΔSpnA were grown until midlogarithmic phase and harvested by centrifugation. The bacterial pellets were washed with a total of 1.0 ml phosphate buffer (20 mM sodium phosphate, 5 mM NaCl, pH 6.0). The pellets were resuspended in 100 μl of 8 M urea and 100 mM ammonium bicarbonate, and the cells were lysed by sonication using Diagenode Bioruptor Plus (4 °C, 15 s on, 15 s off, 40 cycles). The cell debris was removed by centrifugation, and the lysed cells were reduced, alkylated, and trypsin digested for mass spectrometry as described later.

### Preparation and staining of heat-killed bacteria

*S. pyogenes* BMJ71 was grown to midlogarithmic phase at which point the bacteria were washed and concentrated 10-fold in PBS. The bacterial suspensions were transferred to low-bind 1.5 ml tubes (Eppendorf) and left on ice for 15 min. The bacteria were then moved to a shaking heat-block, preheated to 80 °C for 5 min, and transferred directly back to ice for another 15 min. Centrifugation steps to wash or change buffers were all done at 6000 rcf for 5 min. To reduce photobleaching, all stainings were done protected from light. The bacteria were stained with 5 μM Oregon Green 488-X succinimidyl ester (Invitrogen) at 37 °C for 30 min under gentle rotation. The bacteria were subsequently washed in 500 μl sodium carbonate buffer (0.1 M, pH 9.0) necessary for the staining with the pH-sensitive dye CypHer5E (General Electric). The bacteria were then centrifuged, and the final staining volume was reduced to 40 μl whereby the CypHer 5E concentration was 50 μg/ml. Staining was performed at room temperature for 1 h before the bacteria were finally washed in 1 ml of PBS before they were resuspended in a final volume of 1 ml of sodium media (5.6 mM glucose, 127 mM NaCl, 10.8 mM KCl, 2.4 mM KH_2_PO_4_, 1.6 mM MgSO_4_, 10 mM Hepes, 1.8 mM CaCl_2_; pH adjusted to 7.3 with NaOH).

### Proteins, antisera, reagents, and binding assay

Human IgG and serum albumin were purchased from Sigma. The IgG-binding domain of protein G was from GE Healthcare. Rabbit antiserum against recombinant SpnA was generated by BioGenes GmbH, and rabbit antiserum against IdeS was generated by Agriserum. Horseradish peroxidase (HRP)–conjugated protein G and affinity-purified rabbit IgG antibodies against the cell wall carbohydrate antigen specific for *S. pyogenes*, IgG *Strep*A, were purchased from Bio-Rad. Proteins (20 μg; 1 mg/ml in PBS) were radiolabeled with ^125^I using the IODO-BEAD iodination reagent (Pierce) as described by the manufacturer. Binding of radiolabeled proteins to the surface of *S. pyogenes* was performed as previously described (5). Briefly, BMJ71 bacteria from overnight culture were washed with 20 mM sodium phosphate buffer, 5 mM NaCl + 0.05% Tween-20 and varying pH (6.0; 6.5; 7.0 or 7.5) or with 20 mM sodium phosphate buffer, + 0.05% Tween-20, pH 6.0 and varying concentration of NaCl (5 mM; 50 mM or 150 mM). The bacteria were diluted to 2 × 10^9^ colony-forming unit (cfu)/ml in the various buffers, and 200 μl bacterial solution was then incubated with radiolabeled IdeS (0.2–0.5 μg) for 30 min at room temperature. Following a washing step with the respective buffer (see aforementioned), bacteria-bound radioactivity was determined in a γ-counter.

### Cultivation of THP-1 cells

The human monocytic cell line THP-1 was obtained from American Type Culture Collection. The cells were grown in suspension in RPMI1640 medium (Gibco) supplemented with 10% fetal bovine serum (Gibco) at 37 °C and 5% CO_2_. They were continuously kept at a cell concentration between 5 × 10^5^ and 10^6^ to avoid Fc receptor downregulation.

### Phagocytosis assay

The phagocytosis experiments were performed using persistent association normalization (27). Prior to opsonization, the prestained bacteria were sonicated for up to 5 min (VialTweeter; Hielscher) to disperse any large aggregates of bacteria. Sonication was deemed sufficient when clump dispersal was confirmed by microscopy. Staining as well as bacterial count (events/μl in the FITC gate) was assessed by flow cytometry (CytoFLEX; Beckman Coulter). The pH responsiveness of CypHer5E was tested by measuring the bacterial fluorescent staining in the allophycocyanin (APC) channel before and after the addition of 1 μl of sodium acetate (3 M, pH 5.0) to 100 μl of bacterial suspension. The presence of an acid-induced shift in fluorescence indicated successful staining. On the day of experiments, the appropriate number of bacteria were opsonized to suit each experiment. The opsonization with the intact and IdeS-cleaved IgG *Strep*A antibody as well as Xolair was performed at 37 ⁰C for 30 min. For experiments with a variable MOP, serial dilutions of the opsonized bacteria were made and used to incubate with the THP-1 cells. By gating on the leukocyte population, specifically on single cells, we were able to group the cells into those associated with bacteria (FITC positive) and with internalized bacteria (FITC and APC positive). THP-1 cells were counted with a Bürker chamber and resuspended in sodium medium. The concentration of THP-1 cells was adjusted to 2000 cells/μl (100,000 cells per well). The cells were then added to the 96-well plates previously prepared with varying concentrations of previously opsonized bacteria (MOP) or with different antibody concentrations. Finally, 50 μl of THP-1 cells were added on ice resulting in a final phagocytic volume of 150 μl. After a 5 min incubation on ice, the plate was directly transferred to a shaking heating block set to 37 °C while being protected from light. Phagocytosis was stopped by putting the samples on ice for at least 15 min before data acquisition. Four experiments were performed to assess the differences between association curves.

Flow cytometric acquisition was performed using a CytoFLEX with 488 nm and 638 nm lasers and filters 525/40 FITC and 660/10 APC. Threshold was set at FSC-H 70,000 for phagocytosis and for bacteria FSC-H 2000 and SSC-H 2000. Gain was set to 3 for FITC and 265 for APC. Acquisition was set to capture at least 5000 events of the target population with a velocity of 30 μl/min taking approximately 30 min to assess all samples. Throughout the data acquisition, the 96-well plate was kept on an ice-cold insert to inhibit further phagocytosis.

### Construction of a *spnA* deletion mutant in the BMJ71 strain

A *spnA* knockout mutant was generated by replacing the *spnA* gene (locus tag Spy_0747) with the chloramphenicol acetyltransferase (CAT) gene. A 1014 bp region upstream and a 907 bp region downstream of *spnA* was amplified from *S. pyogenes* AP1 genomic DNA by PCR using primers A, B and C, and D listed in Table S1. BamHI and SacI restriction sites were included in primers A and D, respectively. Overlap with the CAT gene was included in primers B and C. The CAT gene was amplified from cloning vector pDC123 (44). The DNA fragments were joined by overlap extension PCR using primers A and D (Table S1) generating a 2581 bp fragment, which was cloned into the BamHI and SacI sites in pFW13 (45), generating plasmid pSE1.

About 100 μl electrocompetent BMJ71 was transformed with 25 μg pSE1 using a GenePulser (Bio-Rad) at 2.3 kV mm^−1^, 3 μF capacitance, and 800 Ω resistance. The bacteria were recovered in rich THY media (TH + yeast extract; Difco) for 2 h at 37 °C before plating on THY/Km (kanamycin; 150 μg/ml) and grown for 48 h at 37 °C. For growth of BMJ71, all THY broth and plates contained tetracycline (5 μg/ml). Colonies were screened for single recombination event, and two clones were chosen for further progress. The two clones were grown overnight in THY/Km, and from there, 100 μl bacteria were transferred into 8 ml THY every day. At each passage, bacteria were diluted 10^3^ to 10^5^ and spread in duplicates on THY/Cml (chloramphenicol; 2 μg/ml) agar and grown 48 h at 37 °C. Colonies were patched on THY/Km and THY/Cml and Cml^R^; Km^S^ colonies were analyzed with PCR for double recombination, and the gene deletion was confirmed by sequencing with primers *spnA* Out F and R (Table S1).

### Cloning, protein expression, and purification of SpnA and IdeS

ORF encoding spnA (National Center for Biotechnology Information NP_268972.1, hypothetical protein Spy_0747) was expressed and purified at the Novo Nordisk Foundation Center for Protein Research. For recombinant expression of the SpnA protein, the DNA sequence, corresponding to residues Val25 to Leu876 (95 kDa), was introduced into the expression vector pNIC-Bsa4 using ligation-independent cloning (46, 47). The resulting *expression* constructs contained a His-tag capture sequence and a tobacco etch virus (TEV) protease cleavage site at the NH_2_ terminus preceding the expressed protein. *Escherichia coli* cells were cultured in terrific broth medium with 50 μg/ml Km. Protein expression was induced with 0.5 mM IPTG, and cell growth was continued for 18 h at 18 °C. Harvested bacterial pellets were suspended in lysis buffer (100 mM Tris, pH 8.0, 300 mM NaCl, 10 mM imidazole, 0.5 mM Tris(2-carboxyethyl)phosphine) complemented with EDTA-free protease inhibitor and ruptured using a French press cell disruptor. Cell debris and membrane components were removed by centrifugation at 40,000*g* for 30 min. Purification of the protein was performed in two steps with the first step employing immobilized metal ion affinity chromatography on an ÄKTA express system (GE Healthcare) at 4 °C with the HiTrap chelating columns. The purified protein was thereafter subjected to treatment with TEV protease to remove the His tag. The final purification was performed by reverse-phase chromatography on a Dionex Ultimate 3000 HPLC system equipped with a preparative C18 HPLC column (Gemini NX; Phenomenex). The protein was eluted by a linear gradient of 10 to 60% acetonitrile, flash-frozen, and lyophilized. The purity and monodispersity of the recombinant protein were confirmed by SDS-PAGE and mass spectrometry.

The *S. pyogenes* ORF encoding for IdeS (UniProt ID: Q7DAM2) was cloned, expressed, and purified at the Lund Protein Production Platform (LP3). The ORF, including a 6xHis-HA-StrepII-TEV (histidine–hemagglutinin–StrepII–TEV protease recognition site) tag on the C terminus, was ordered as a synthetic construct from Genscript and cloned into the pET-26b(+) vector. IdeS was expressed in LB broth supplemented with 50 μg/ml Km at 18 °C in *E. coli* TUNER (DE3) cells, with expression being induced with 1 mM IPTG at an absorbance of 0.8 at 600 nm. The cells were harvested after 21 h and resuspended in phosphate buffer (50 mM sodium phosphate [pH 8.0], 300 mM NaCl, and 20 mM imidazole) supplemented with EDTA-free Complete Protease Inhibitor tablets (Roche). The cells were lysed using a French pressure cell at 18,000 psi. The lysate was cleared *via* ultracentrifugation (Ti 50.2 rotor, 244,000*g*, 60 min, 4 °C) and subsequently passed through a 0.45 μm filter prior to loading on a HisTrap HP column (GE Healthcare). The column was washed with 20 column volumes of phosphate buffer, and bound protein was eluted using a gradient of 0 to 500 mM imidazole in phosphate buffer. Fractions containing the desired protein were pooled. The purified IdeS was mixed with TEV at an TEV:IdeS mass ratio of 1:12, DDT was added to a final concentration of 1 mM, and the digestion was done overnight at 16 °C. After the TEV protease digestion, the TEV-cleaved IdeS was purified on a HisTrapHP column as aforementioned with the flowthrough fraction collected. IdeS was further purified by size-exclusion chromatography using a Hi load 26/600 Superdex 200 pg column (GE Healthcare) in 1× PBS, pH 7.4.

### IdeS-mediated antibody cleavage

IgG *Strep*A (100 μg) was incubated with IdeS (final concentration of 3 μg/ml) overnight at 37 °C while shaking gently. To ensure for thorough cleavage of the antibody, 10 μg was analyzed by SDS-PAGE (4–20% gradient gel), together with uncleaved antibody and a monoclonal IgG antibody (Xolair, omalizumab) against immunoglobulin E used as a nonbinding IgG control antibody. The gel was then stained with GelCode Blue protein stain (Thermo Scientific) for 30 min whereafter the gel was destained in deionized water overnight before imaging in a Bio-Rad ChemiDoc system.

### Coupling of SpnA to Sepharose

SpnA (20 mg) was dialyzed against coupling buffer (0.1 M NaHCO_3_ [pH 8.3] + 0.5 M NaCl). One gram of cyanogen bromide–activated Sepharose (cyanogen bromide 4B; GE Healthcare) was resuspended and washed with 1 mM HCl followed by a final washing step with coupling buffer. SpnA was added and allowed to bind under rotation for 3 h at room temperature. Thereafter, the Sepharose was washed with coupling buffer and incubated with blocking buffer (0.1 M Tris [pH 8.0]) for 2 h at room temperature. Finally, the SpnA-Sepharose was washed with alternating pH (0.1 M sodium acetate [pH 4.0] + 0.5 M NaCl and 0.1 M Tris [pH 8.0] + 0.5 M NaCl) followed by a final washing step with 20 mM sodium phosphate buffer [pH 6.0] + 5 mM NaCl.

### Preparation of anti-SpnA F(ab’)_2_ fragments

Rabbit anti-SpnA antiserum (4 ml) was applied to a protein G-Sepharose column of 2 ml in PBS (protein G-Sepharose from GE Healthcare). Following extensive washing with PBS, bound IgG was eluted with 0.1 M glycine–HCl buffer (pH 2.0). Fractions of 3 ml were collected, and the pH in the fractions was raised to 7.5 with 1 M unbuffered Tris solution. IgG-containing fractions were applied to a SpnA-Sepharose column (0.6 ml) in PBS. After extensive washing with PBS, bound IgG was eluted as described previously and subjected to IdeS cleavage using a FragIT kit as described by the manufacturer (Genovis AB). Following cleavage, the anti-SpnA F(ab’)_2_ fragments were subjected to buffer exchange to 20 mM sodium phosphate (pH 6.0) + 5 mM NaCl using microspin concentration filters (Amicon ultra filter; Merck).

### Analysis of SpnA at the surface of BMJ71

*S. pyogenes* BMJ71 bacteria were grown in TH broth to stationary phase, washed, and diluted to 2 × 10^9^ cfu/ml in PBS. A 200 μl bacterial solution was incubated with 10 μl of preimmune serum or anti-SpnA antiserum (undiluted, diluted 1:10, 1:100, and 1:1000) for 1 h at room temperature. Then the bacteria were washed with PBS, resuspended in 200 μl of PBST (PBS + 0.05% Tween-20), and incubated with ^125^I-labeled protein G (0.2–0.5 μg) for 30 min at room temperature. Following a washing step with PBST, bacteria-bound radioactivity was determined in a γ-counter.

### SDS-PAGE, Western blot, and slot binding

Proteins were separated by 4 to 20% SDS-PAGE using the Laemmli buffer system. Gels were stained by PAGE-Blue (Thermo Scientific) or subjected to Western blot. Proteins were also directly applied to polyvinylidene difluoride membranes using a Milliblot-D system (Millipore). For incubation with radiolabeled SpnA, the membrane was blocked in Tris-buffered saline (0.05 M Tris–HCl, pH 7.5, 0.15 M NaCl) containing 3% bovine serum albumin (Sigma) and incubated with ^125^I-labeled SpnA (0.4–0.8 μg/ml in the same buffer) for 3 h at room temperature or at 4 °C overnight. The membrane was then washed with Tris-buffered saline containing 0.05% Tween-20, and binding was visualized using the Fuji FLA-3000 Imaging system. For incubation with antibodies, membranes were blocked in PBST containing 5% dry milk powder (blocking buffer), incubated with primary antibodies (1:1000 dilution) in blocking buffer for 30 min at 37 °C, washed with PBST, and incubated with HRP-conjugated protein G (1:3000 dilution; Bio-Rad), or HRP-conjugated goat anti-rabbit IgG (1:3000 dilution; Bio-Rad) in PBST. Following a washing step with PBST, the bound antibodies were detected by chemiluminescence.

### Bacterial growth curves, analysis of IdeS and SpnA expression

The *S. pyogenes* BMJ71 and ΔSpnA strains were grown in TH-broth overnight at 37 °C, 5% CO_2_. From these cultures, 0.4 ml was transferred to 8 ml fresh TH broth, and the absorbance at 620 nm was measured every hour. Bacterial aliquots of 0.4 ml were also transferred to 10 tubes, each containing 8 ml fresh TH broth, and cultivated for 0, 1, 2, 3, 4, 5, 6, 7, 8, and 9 h. At each time point, the growth medium was collected by centrifugation and sterile filtered. One milliliter of the growth medium was then precipitated with trichloroacetic acid (5% final concentration) and analyzed by Western blot as described previously. The bacterial cells from overnight, 5 h, and 9 h cultures were washed with 20 mM sodium phosphate buffer (pH 6.0) + 5 mM NaCl, resuspended in SDS loading buffer, heated at 85 °C for 10 min, and the resulting supernatant was analyzed by Western blot for expression of SpnA.

### Biacore

Surface plasmon resonance spectroscopy (Biacore T100; GE Healthcare) was used to determine the association and dissociation rate constants for the interaction between SpnA and IdeS. SpnA was immobilized on a CM5 sensor chip (GE Healthcare) using amino coupling and following instructions provided by the manufacturer. As running buffer, Hepes buffer (100 mM Hepes, 150 mM NaCl, 0.05% surfactant P20, pH 7.4) was used. The immobilization of SpnA resulted in 7247 resonance units. IdeS was injected at different concentrations (10, 20, 40, 80, and 160 μM) at a flow rate of 10 μl/min, with a contact time of 60 s (refers to sample injection, corresponds to association) and a dissociation time of 120 s. Glycine–HCl buffer (10 mM, pH 2.5) was used for regeneration. Binding affinities were calculated from rate constants obtained from fitting the data to a 1:1 Langmuir binding model.

### Flow cytometry

IdeS (1 mg) was labeled with Alexa-Fluor 633 labeling kit (Molecular Probes) according to instructions of the manufacturer. Stationary phase BMJ71 bacteria were washed with 20 mM sodium phosphate buffer (pH 6.0) + 5 mM NaCl and resuspended to a concentration of 2 × 10^7^ cfu/ml. Suspensions of 100 μl were incubated with 2 μl labeled IdeS (4 mg/ml) for 30 min at room temperature in the dark. Thereafter, 400 μl buffer was added (unwashed samples), and the samples were analyzed. Alternatively, samples were centrifuged for 10 min at 1000*g*, resuspended in 500 μl buffer (washed samples), and analyzed. All samples were analyzed on a BD Accuri flow cytometer using logarithmic acquisition. For unwashed samples, 10,000 events were recorded, and for washed samples, 3000 events were recorded. The results were analyzed using Accuri C6 software (BD Bioscience).

### Gel filtration and IdeS cleavage of IgG

SpnA (600 μg) was incubated with IdeS (200 μg) for 30 min at room temperature (proteins were in 20 mM sodium phosphate buffer [pH 6.0] + 5 mM NaCl) and then subjected to separation on a Superose 12 10/300 GL gel filtration column (GE Healthcare) equilibrated with the aforementioned buffer. Fractions of 0.25 ml were collected, and the absorbance at 280 nm was measured using a Beckman spectrophotometer. SpnA (600 μg) or IdeS (400 μg) alone was also incubated for 30 min at room temperature in the aforementioned buffer and then separated on the Superose 12 column. Fractions were analyzed by SDS-PAGE or by slot binding assays using anti-IdeS (1:1000 dilution) as a probe. Fractions from separation of SpnA–IdeS complexes were also tested for cleavage of IgG. Human polyclonal IgG (3 μg) was incubated with 10 μl of the fractions for 1 h at 37 °C. Cleavage was analyzed by SDS-PAGE.

### Structured illumination super-resolution fluorescence microscopy

Midlogarithmic cultures (absorbance at 620 nm = 0.4–0.45) of BMJ71 and ΔSpnA were prepared. The bacteria were then washed with 20 mM sodium phosphate buffer (pH 6.0) + 5 mM NaCl, resuspended to a concentration of 2 × 10^9^ cfu/ml, and further diluted to 2 × 10^7^ cfu/ml. To 100 μl of bacterial solution, 6 μl of Alexa Fluor 647-conjugated IdeS (6 μg/ml), and/or 6 μl Alexa Fluor 488-conjugated anti-SpnA F(ab')_2_ fragments (0.2 μg/ml) were added. Samples were incubated for 1 h at room temperature in the dark. Following incubation, 400 μl of the aforementioned buffer was added, and samples were fixed with 250 μl 16% paraformaldehyde for 45 min at room temperature. Bacteria were washed with PBS, resuspended in 50 μl PBS, and 3 μl was pipetted on to a glass slide. About 3 μl Gold antifade mountant was added, and a #1.5 coverslip was placed on top. The samples were allowed to cure overnight before imaging.

For image acquisition, a Nikon N-SIM E system with a 100× oil objective (numerical aperture = 1.49) coupled to a Hamamatsu Orca Flash 4.0 camera was used to acquire images. 3D SIM images were acquired for two channels: 488 nm laser for AF488-anti-SpnA F(ab’)_2_ fragments and 640 nm laser for AF647-conjugated IdeS. The SIM images were reconstructed with the NIS-elements AR algorithm for reconstruction. For colocalization analysis, the images were background-subtracted and processed in ImageJ (open source.imagej.net) with the Coloc2 plugin.

### Analysis of IgG cleavage at the bacterial surface

Midlogarithmic phase BMJ71 bacteria were washed with 20 mM sodium phosphate buffer (pH 6.0) + 5 mM NaCl. One milliliter bacterial suspensions (2 × 10^9^ cfu/ml) were incubated with rabbit IgG antibodies (50 μg) specific for *S. pyogenes* (IgG *Strep*A) or with IdeS (50 μg) for 1 h at room temperature under rotation. The samples were centrifuged for 1 min at 10,000*g*, and the supernatants were recovered and sterile filtered. The bacterial cells were washed three times with 20 mM sodium phosphate buffer (pH 6.0) + 5 mM NaCl and thereafter resuspended in 1 ml of the same buffer. Buffer or IdeS (50 μg) was added to bacteria incubated with IgG *Strep*A, and to bacteria incubated with IdeS, the IgG *Strep*A antibodies were added. Samples were then incubated at 37 °C for 1 h under rotation. Following centrifugation at 10,000*g*, the supernatants were recovered and sterile filtered. The six supernatants were precipitated with trichloroacetic acid (final concentration of 5%) and subjected to analysis by SDS-PAGE.

In another set of experiments, the BMJ71 and *spnA* mutants were grown overnight in TH. From each culture, 400 μl was transferred to new tubes with 8 ml TH, and the cultures were grown to absorbance approximately 0.4. The bacteria were washed with 20 mM phosphate buffer (pH 6.0) + 5 mM NaCl and resuspended to 1% solution. One milliliter bacterial solution from each strain was incubated with rabbit anti-strepA IgG (50 μg). The samples were incubated for 1 h under rotation at room temperature. The supernatants were collected, sterile filtered, and stored at −20 °C and analyzed in Western blots. Membranes were blocked in 5% dry milk at room temperature, incubated with HRP-conjugated goat anti-rabbit IgG (1:3000 dilution) in PBST for 1 h at room temperature, washed with PBST, and developed with chemiluminescence. The intensity of the bands was analyzed with the Fiji software (open source Fiji.net) as follows: The intensity of the 56 kDa intact heavy chain band of IgG and the 31 kDa IdeS-cleaved heavy chain fragment were measured separately. These numbers (%) were then combined as total intensity, and then the amount of the cleaved 31 kDa band of the total intensity was calculated as exemplified by the experiment below.

**Table:** 

Strain	31 kDa %	56 kDa %	Total intensity %	Cleavage %
BMJ71	51.49	36.95	88.44	58.2
ΔSpnA	48.51	63.06	111.57	43.5

Data were evaluated using unpaired *t* test.

### Sample preparation from mass spectrometry

The samples from the lysed cell fraction of the intact BMJ71 or BMJ71 ΔSpnA strains were denatured with 8 M urea and 100 mM ammonium bicarbonate, and the cysteine bonds were reduced with 5 mM Tris(2-carboxyethyl)phosphine (37 °C for 60 min) and alkylated with 10 mM iodoacetamide (22 °C for 30 min). Samples were diluted with 100 mM ammonium bicarbonate to a final urea concentration of 1.5 M, and sequencing grade trypsin (Promega) was added for protein digestion (37 °C for 18 h). Samples were acidified (to a final pH 3.0) with 10% formic acid, and the peptides subsequently purified with C18 reverse-phase spin columns according to the manufacturer’s instructions (macrospin columns; Harvard Apparatus). Peptides were dried in a speedvac and reconstituted in 2% acetonitrile and 0.2% formic acid prior to mass spectrometric analyses.

### Liquid chromatography tandem mass spectrometry

The analysis of the peptide samples was performed on an Eclipse mass spectrometer connected to an ultra–high-performance Ultimate3000 liquid chromatography system (Thermo Scientific). The peptides were then separated on a Thermo EASY-spray column (Thermo Scientific 25 cm column, column temperature of 45 °C) and operated at a maximum pressure of 900 bar.

For the analysis of the soluble and lysed cell fraction of the intact BMJ71 or BMJ71 ΔSpnA strains, a nonlinear gradient of 2 to 25% of 80% acetonitrile in aqueous 0.1% formic acid was run for 100 min followed by 25 to 40% of 80% acetonitrile in aqueous 0.1% formic acid for 20 min at 300 nl^−1^ min. One full MS scan (resolution of 12,000 for a mass range of 350–1400 *m/z*) was followed by MS/MS scans (resolution of 15,000). The cycle time was 3 s. The precursor ions were then isolated with a 1.6 *m/z* isolation window and fragmented using higher-energy collisional-induced dissociation at a normalized collision energy of 30. The dynamic exclusion was set to 45 s.

### Proteomics data analysis

The mass spectrometry data were analyzed in Proteome Discoverer 2.5 (Thermo Scientific) against the *S. pyogenes* UniProt proteome (proteome ID: UP000000750). Fully tryptic digestion was used allowing two missed cleavages. Carbamidomethylation (C) was set to static and protein N-terminal acetylation and oxidation (M) to variable modifications. Mass tolerance for precursor ions was set to 10 ppm and for fragment ions to 0.02 Da. The protein false discovery rate was set to 1%. Proteins identified by two or more unique peptides were considered as relevant, and others were discarded. The label-free quantification intensities were log2 transformed and mean normalized in Perseus (version 2.0.2.0) (https://maxquant.net/perseus/) for the generation on volcano plots (for volcano plots, false discovery rate = 0.01). For the generation of boxplots in GraphPad Prism (GraphPad Software, Inc), the label-free quantification intensities were sum normalized. Missing values were imputed from the Gaussian distribution (width: 0.3, downshift: 1.8).

### Statistical analysis

Prism 8 was used to perform statistical analysis, and significance was tested using one-way ANOVA or unpaired *t* test. Mander's and Pearson's correlation was used for image colocalization analysis (48).

## Data availability

The mass spectrometry data have been deposited to the ProteomeXchange (49) consortium *via* the MassIVE partner repository (https://massive.ucsd.edu/) with the dataset identifier PXD045490. Dataset access for reviewers: Username: MSV000092893_reviewer; Password: 16e5∗∗Spn4.

## Supporting information

This article contains supporting information.

## Conflict of interest

The authors declare that they have no conflicts of interest with the contents of this article.
