# Supplementary material for: IdeS, a secreted proteinase of Streptococcus pyogenes, is bound to a nuclease at the bacterial surface where it inactivates opsonizing IgG antibodies
Source: J Biol Chem. 2023 Oct 12;299(11):105345. doi: 10.1016/j.jbc.2023.105345 (PMC10654033; doi:10.1016/j.jbc.2023.105345)
Supplement: Supporting information [file mmc1.docx]

**Supporting Information**

**IdeS, a secreted proteinase of *Streptococcus pyogenes,* is bound to a nuclease at the bacterial surface where it inactivates opsonizing IgG antibodies.**

Inga-Maria Frick^*^, Lotta Happonen, Sebastian Wrighton, Pontus Nordenfelt, and Lars Björck^*^

**Figure S1**

**Figure S2**

**Figure S3**

**Figure S4**

**Table S1**
