# Supplementary material for: IdeS, a secreted proteinase of Streptococcus pyogenes, is bound to a nuclease at the bacterial surface where it inactivates opsonizing IgG antibodies
Source: J Biol Chem. 2023 Oct 12;299(11):105345. doi: 10.1016/j.jbc.2023.105345 (PMC10654033; doi:10.1016/j.jbc.2023.105345)
Supplement: Supporting Tables S1 [file mmc2.docx]

**Supporting Table 1.**

**Primers used for construction of a Spna mutant in the BMJ71 strain**

**Name Sequence**

SpnAUpF BamH1(A)

5’-GATCGGATCCTTACGTCTGTGTAGGATTTG-3’

SpnAUpR + cat (B) 5’-CCAGTGATTTTTTTCTCCATGATATCTCCT TTTAATGTGAT-3’

SpnADownF + cat (C) 5’-GCAGGGCGGGGCGTAACGATTTGAAGAATT

GCAGTTC-3’

SpnADownR SacI (D) 5’-GATCGAGCTCGGCCACTCCATATGGTAC-3’

Cat For 5’-ATGGAGAAAAAAATCACTGGAT-3’

Cat Rev 5’-TTACGCCCCGCCCTG-3’

*spnA* out For 5’-GCTGTCTTGTTCGCTATCAGG-3’

*spnA* out Rev 5’-GACAACATACAAGGCAGCTAC-3’

**Primers A and B:** Amplified upstream fragment of deletion construct. Restriction site is

underlined

**Primers C and D:** Amplified downstream fragment of deletion construct. Restriction site is

underlined

**Cat (chloramphenicol acetyltransferase)**

**Cat For (forward) + Cat Rev (reverse):** Amplified CAT gene

***spnA* out For + *spnA* out Rev:** Sequencing primers
