# Supplementary material for: IdeS, a secreted proteinase of Streptococcus pyogenes, is bound to a nuclease at the bacterial surface where it inactivates opsonizing IgG antibodies
Source: J Biol Chem. 2023 Oct 12;299(11):105345. doi: 10.1016/j.jbc.2023.105345 (PMC10654033; doi:10.1016/j.jbc.2023.105345)
Supplement: Supporting Figure Legends S1–S4 [file mmc3.docx]

**Supporting Figures Legends**

**Figure S1.**

**SDS-PAGE analysis of IgG *Strep*A, IgG *Strep*A cleaved by IdeS, and Xolair**

IgG StrepA (100 ug) was cleaved by IdeS (final concentration of 3 µg/ml) over-night at 37°C. Intact IgG *StrepA*, cleaved IgG *StrepA* and Xolair (10 μg) were analyzed by SDS-PAGE (gradient gel 4-20%). The gel was stained with GelCode Blue protein stain.

**Figure S2. Analysis of the SpnA mutant strain**

**(A)** BMJ71 and BMJ71ΔSpnA were grown over night in TH broth. A portion of 400 μl from each strain was transferred to a new tube with 8 ml TH and cultivated for 9h. The optical density at 620 nm was measured at indicated time points. **(B)** BMJ71 and BMJ71ΔSpnA were grown over night in TH broth. A portion of 400 μl was transferred to two tubes (for each strain) with 8 ml fresh TH and cultivated for 5h and 9h. Bacteria from the o/n, 5h and 9 h growth were washed with PBS and boiled in SDS-PAGE loading buffer. Samples were analysed by Western blot using antiserum against SpnA (diluted 1:1000) as the probe. **(C)** BMJ71 and ΔSpnA were grown over night in TH broth. A portion of 400 μl was transferred to 10 tubes (for each strain) with 8 ml TH and cultivated for the indicated time points. At each time point the supernatants were collected, sterile filtered and precipitated with TCA. All samples were subjected to Western blot using antiserum against IdeS (diluted 1:1000) as the probe.

**Figure S3. Analysis of IdeS in gel filtration experiments**

**(A)** IdeS was separated on a Superose 12 gel filtration column in 20 mM phosphate buffer pH 6.0 + 5mM NaCl. Fractions of 0.25 ml were collected and the absorbance at 280 nm was measured. **(B)** Ten ul of the fractions from **(A)** were applied in slots to a PVDF membrane probed with anti-IdeS antibodies followed by HRP conjugated IgGFc-binding protein G. **(C)** SDS-PAGE of purified IgG, IdeS, IgG+IdeS and IgG+fractions 37, 53 or 61from the chromatogram of **(A)**. The 31 kDa fragment of IgG heavy chains generated by IdeS cleavage is indicated by the arrow.

**Figure S4. Cleavage of IgG strepA on the surface of BMJ71 and the *spnA* mutant**

The BMJ71 and the *spnA* mutant were grown overnight in TH. From each culture 400 μl was transferred to new tubes with 8 ml TH and the cultures were grown to mid-logarithmic phase. The bacteria were washed with 20 mM phosphate buffer pH 6.0 + 5 mM NaCl and resuspended to 1% solution. One ml bacterial solution from each strain was incubated with rabbit anti-strepA IgG (50 μg) for 1 h under rotation at room temperature. The supernatants were collected, sterile filtered and analyzed with Western blot. Membranes were blocked in 5% dry milk at room temperature, incubated with HRP-conjugated goat anti-rabbit IgG (1:3000) in PBST for 1h at room temperature, washed with PBST and developed with chemiluminescence. The intensity of the 56 kDa band (intact IgG heavy chains) and the 31 kDa IdeS-generated fragment was analyzed separately with the Fiji software. These numbers (%) were then combined as total intensity and then the amount of the cleaved 31 kDa band in percent of total intensity was calculated. Data shown are mean values from five experiments ± S.D. and were evaluated using unpaired T-test.
